# Report on the Progress of Toxicology, in Relation to Medical Jurisprudence, Medical Police, Chemistry, and Pharmacy, for the Years 1843-4

**Published:** 1844-10

**Authors:** Alfred S. Taylor

**Affiliations:** Lecturer on Medical Jurisprudence and Chemistry in Guy's Hospital


PART THIRD.

### Original Reports and Memoirs.

### REPORT ON THE PROGRESS OF TOXICOLOGY,

IN RELATION TO

MEDICAL JURISPRUDENCE, MEDICAL POLICE, CHEMISTRY, AND PHARMACY, FOR THE YEARS 1843-4.

By Alfred S. Taylor,
Lecturer on Medical Jurisprudence and Chemistry in Guy's Hospital.

The object of this paper is to present, in a condensed form, the principal discoveries which have been made in relation to toxicology, during the past year. Many of the facts have been selected from English medical periodicals, as these have a peculiar interest to the British practitioner; but some have been also derived from the two principal medico-legal journals of France and Germany, namely the 'Annales d'Hygiène,' and Henke's 'Zeitschrift der Staatsarzneikunde.' Notices of the more important trials for poisoning that have taken place in Great Britain during this period, are appended. Some cases have necessarily been omitted; the selection being here confined to those facts which appeared to have any practical interest.

### IRRITANT POISONS.

*Cold liquids.* The symptoms produced by cold liquids when swallowed in large quantity, have been often mistaken for those of irritant poisoning, and where death has ensued, this has been ascribed to poison. Facts of this kind, which are of some value in aiding our diagnosis in cases of poisoning, have been hitherto imperfectly observed and but rarely recorded. Mr. Cridland of Chelsea has reported the following case, (Lancet, Oct., 1843,) which occurred in the month of August preceding, at a time when the weather was hot and sultry. A child, aged 9, rose from her bed with her body much heated, and drank off a small cupful of cold water. She immediately fell to the ground in a state of insensibility. Half an hour afterwards, when seen by a medical man, she was unconscious; her skin was cold, the pulse feeble, and the pupils were unaffected by light. There were also convulsive twitchings about the corners of the mouth. She was bled, stimulants were applied, and in about five or six hours she recovered. The effects were here probably due to a shock to the nervous system. Had the nature of the liquid taken been unknown, the symptoms might have been speciously referred to a dose of prussic acid.

In regard to the effects of cold liquids and the medico-legal questions which arise respecting them, the reader may refer to an elaborate paper on the subject by Dr. Guérard, in the 'Annales d'Hygiène' for January 1842, p. 42.

*Mechanical irritants. Sponge* has been regarded as a mechanical irritant capable of producing inflammation and death. Accidents of this kind have been observed in young infants. In May last, Dr. Chowne reported to the Westminster Medical Society, a case which, however, shows that this substance sometimes exerts no action whatever upon the system. An infant, three months and a half old, swallowed a small piece of sponge, which had been placed in the nipple of a sucking bottle. A dose of castor oil was given, and the sponge was passed per anum fourteen hours after it had been swallowed. There were no symptoms.

A case has been tried in this country, where a woman was charged with the death of a child by the administration of sponge.

*Diagnosis of cases of poisoning.* It is well known that there are certain diseases which in their symptoms and rapidly fatal character, closely resemble cases of irritant poisoning. The diagnosis is here of great importance, not merely in relation to treatment, but in regard to the defence which may be set up, should the individual die and a person be charged with the crime of poisoning. In the law-reports, many cases are recorded in which, on trials for arsenical poisoning, the poison not having been detected in the body, the parties charged with the crime have been acquitted, owing to the difficulty of distinguishing them from cholera and acute inflammation of the stomach and bowels. Cases of arsenical poisoning which have occurred in autumn, have been often treated as cases of cholera, and the mistake has been discovered only by an examination of the body or by the confession of the criminal. It has been doubted by some pathologists, whether *gastritis,* enteritis, or peritonitis, diseases which in their symptoms somewhat resemble cases of irritant poisoning, can occur spontaneously or without an obvious and apparent cause. It is certainly rare to hear of cases of idiopathic acute *gastritis* occurring in individuals otherwise healthy, but a well-marked instance of the kind has been lately reported by Mr. Berncastle, (Lancet, March, 1844.) The symptoms were of the usual character; constant vomiting, no diarrhea, and rapid sinking. After death, the stomach was found in a high state of inflammation, but all the other viscera were healthy.

The case of the Queen *v.* Hunter, tried at the Liverpool Spring Assizes, 1843, is worthy of the attention of medical witnesses in relation to the appearances produced by irritant poisons, and the diseases above referred to. In this case, a woman was charged with having poisoned her husband by arsenic. The medical evidence rested chiefly on the symptoms and post-mortem appearances, for no arsenic was discovered in the body. The mucous membrane of the stomach and intestines was found, throughout its whole extent, exceedingly inflamed and softened. The medical witnesses for the prosecution referred this condition to the action of arsenic; those for the defence considered that it might be owing to idiopathic gastro-enteritis, independently of the exhibition of any irritant. The circumstances of the case were very suspicious; but the prisoner was acquitted, not merely on account of the variance in the medical evidence, but from the absence of positive proof of poison, i.e. its detection by chemical analysis. This generally weighs much with a court of law, although it is well known that arsenic cannot always be detected in the body of a person who has undoubtedly died from a large dose of that substance. It is right to state, as a warning to medical witnesses, that the judge who tried the case expressed regret that, on the non-discovery of poison in the contents of the stomach and intestines, the soft parts of the body (the muscles) had not been examined according to the processes lately suggested by Orfila. (See the published reports of the case by Mr. Holland and Mr. Dyson.)

*Perforation of the stomach and intestines.* This disease often leads to death under circumstances resembling irritant poisoning. Any well-ascertained facts, therefore, connected with the subject are of some interest to the medical jurist. In November 1843, Dr. Seymour reported two cases of death from perforation of the stomach to the Medico-Chirurgical Society. The subjects of the two cases were, as usual, females: one about the age of twenty, the other twenty-five. Perforation of the stomach generally proves fatal in from eighteen to thirty-six hours, by inducing peritonitis; but these cases were remarkable in the circumstance, that one of the females lived ten days and the other a fortnight after the probable time of perforation. On inspection, the ulcers in the stomach were found to communicate with cysts.

There is one insidious form in which perforation of the intestines may present itself, and simulate irritant poisoning, although the real cause of death (peritonitis) will be immediately apparent on inspection. I allude to the formation of an aperture by ulceration, in the extreme end of the appendix vermiformis cæci owing to the pressure produced by some calculous concretion. Two cases have been communicated to me, both of which occurred in young men apparently in good health; and they proved speedily fatal under the usual symptoms. In both cases the perforation was produced by a hard substance lodged at the end of the appendix. These substances were sent to me for analysis. In one case the calculus was about the size of a large pea, and it consisted of inspissated mucus, biliary matter, and a large quantity of carbonate of lime; in the other the calculus was smaller, but similar in composition. For the detection of such cases, a careful inspection is required, since, both the aperture and the calculus being small, the source of the fatal effusion might be easily overlooked.

It is not often that cases of narcotic poisoning are mistaken for those of disease, but still, where the facts are intentionally concealed or wilfully misrepresented by the criminal administrator, any medical practitioner is liable to be misled. The diseases which in their symptoms and course resemble narcotic poisoning, are generally well-marked in their characters during life, or in the post-mortem appearances found on inspection. A case has just been tried at the Lincoln assizes, (July, 1844,) which shows that a crafty criminal may easily deceive a medical practitioner, and that the coroner's inquest, as it is at present conducted, is not fitted to detect these secret cases of poisoning. In this case, a confession was made; but how many instances escape detection for want of a confession on the part of a criminal, it is impossible to conjecture. An inspection of a body is not required by many coroners unless there are strong circumstances for suspicion in the shape of public rumour; but in respect to criminals, who have well calculated their plans, these circumstances are not likely to come to light *except from a post-mortem inspection, and an analysis of the contents of the viscera.** It does not appear that any inquisition was held or inspection made in the case alluded to, until some time after the bodies of the deceased had been interred, and then it was too late. A woman was charged with the murder of three children by poisoning one of them with arsenic, and the other two with opium. She pleaded guilty, and confessed the manner in which the crime was perpetrated. She had succeeded in poisoning two of the children without being detected, although suspicion was so strong that she was tried, but acquitted, at the previous assizes, on the charge of having poisoned one of them. In the third case, she admitted having secretly given to the deceased, (her own infant,) about three weeks old, a teaspoonful of laudanum. The child was soon afterwards seized with convulsions; a medical practitioner was sent for, who, deceived by the statement of the woman, treated it as a case of ordinary convulsions in children, and ordered a warm bath. The child died in about twenty hours, continuing, according to the prisoner's statement, in convulsions during the greater part of that time. No suspicion appears to have been entertained of the real cause of death, and the case would probably have remained undiscovered, but for the prisoner's confession. It is remarkable that this child survived so long; the woman, however, prevaricated as to the quantity of laudanum which she gave it, therefore it is difficult to draw any conclusion from her statement, except that the deceased was actually poisoned by opium. (The Queen *v.* Joyce.)

* In one case just referred to me from the country (Sept. 1844), the jury, under the direction of the coroner, returned a verdict of death from poison ("misadventure,") while the stomach of the deceased was in my custody, and *before it had even been opened, or the seals of the vessels containing it had been broken!* In another, now under examination, in which there is the very strongest reason to suspect death from poison administered by a quack, the coroner and jury declined waiting for an analysis (not yet completed) of the contents of the stomach, although strongly advised by the medical witness who inspected the body,—and returned a verdict of "*natural death.*" It is absurd to talk of coroners being legally responsible for such an abuse of their office. The juries, who act under them, are very unlikely to be the complaining parties; the country magistrates, even if they were made acquainted with the facts, are not likely to conceive the necessity for such an additional charge to the county rates as these chemical analyses would often entail; and lastly, where is the medical practitioner who can afford the time and expense of enforcing legal proceedings against a county coroner, even supposing him to be desirous of filling such an invidious office as that of public prosecutor? In the *theory* of the English law, "there is no wrong without a remedy;" in the *practice,* the wrongs are numerous, and the remedies might as well not exist, for they are in many instances quite unattainable!

*Phosphorus.* It is not often that we hear of cases of poiscning by phcsphorus or its compounds, but the following instance has been reported by Mr. Shephard of Stonehouse. (Lancet, Dec. 1843.) A child, between two and three years of age, had been caught in the act of sucking and swallowing the heads of lucifer matches. Two days afterwards she appeared unwell, there was some feverish excitement, but no active symptoms. The bowels were open, but the child did not suffer from pain, vomiting, or diarrhea. Five hours after she was first seen, she became violently convulsed, and she died three hours afterwards. On inspection, a quantity of mucus mixed with blood, of a coffee-ground colour, was found in the stomach. The mucous membrane of the organ was very vascular throughout, and for the space of about two inches it had a florid red colour, and was covered with mucus. There were no less than ten invaginations in the small intestines, many of which included from two to three inches of intestine, which was inflamed at the invaginated parts. There was no appearance of strangulation, and the bowels were empty. The medical opinion given at the inquest was that phosphorus, in a finely divided state, was the cause of death, and a verdict was returned accordingly.

*Arsenic.* The subject of arsenical poisoning continues to receive much attention from toxicologists, and suggestions are continually being made for improving the numerous processes which are now employed for the detection of this poison. Among the late announcements on this subject, is a new method of employing Marsh's test, suggested by Mr. Ellis of University College. Instead of burning the arsenuretted hydrogen 'gas, and receiving the deposit of metallic arsenic on cold plates of glass, porcelain, or metal, Mr. Ellis proposes to decompose the gas and deprive it of arsenic by passing it over metallic copper, or one of the oxides of that metal. In passing arsenuretted hydrogen gas, generated in the usual way, over slips of metallic copper gently heated, he found that this metal acquired a gray coating of arsenic similar to that procured in the application of Reinsch's test. (See Report, No. XIX of this Journal, p. 275.) If the surface of the copper were oxidated, then no heat was required for the decomposition. Metallic arsenic was immediately deposited when a current of the gas was passed over the oxidized surface, owing to a decomposition of the gas and the oxide. This discovery led Mr. Ellis to employ at once oxide of copper instead of the pure metal, and he found that during this decomposition, the black oxide became gray, and the red oxide black. The well-marked change of colour, rendered the use of the latter preferable, as it at once served to indicate the progress of the decomposition. The arsenuretted hydrogen gas is first dried by passing it over fused chloride of calcium, contained in a bulb attached to Marsh's apparatus; and it is then allowed to pass over the oxide of copper in a tube, the current of gas being controlled by a stop-cock; for if it should pass too rapidly, it escapes decomposition. No heat is required for the change;—the oxide of copper absorbs the gas as it slowly passes and becomes heated;—water is produced, and the arsenic is retained by the oxide, either as arsenuret or arsenite of copper. By occasionally igniting the jet at the end of the tube, it may be seen whether the arsenic has been entirely removed from it or not, and the process arrested or continued accordingly. When the oxide is saturated with arsenic, it may be removed and gently heated in a small reduction tube; a brilliant ring of octohedral crystals of arsenious acid will be thereby obtained. These crystals are equally produced in an atmosphere of carbonic acid or hydrogen, showing that the arsenic must either have existed as arsenious acid in the compound, or have derived its oxygen from the oxide of copper. When a sublimate of arsenious acid has been thus procured, it may be dissolved in water and tested in the usual way.

While it must be admitted that this is a very ingenious process, it cannot be denied that it is open to all the objections to which Marsh's test, as it is commonly employed, is liable. This, however, is apparent, that it is far less liable to lead to a loss of arsenic; and thus, where the quantity of poison is extremely small, we may be more certain of obtaining evidence of its presence by using the oxide of copper, than by merely burning the gas, and exposing a cold plate to the flame. Small sublimates are in this way often dissipated, and a quantity of arsenic is invariably lost at each time the gas is ignited. The objections to the plan are that additional apparatus with very careful manipulation is required. It does not appear to be in any respect preferable to Reinsch's test, and in delicacy is far inferior to the plan proposed by Dr. Clark, of passing the arsenuretted hydrogen gas into a solution of nitrate of silver.

Dr. R. Fresenius of Giessen, has lately caused to be read before the Chemical Society, a series of elaborate papers, since published in the 'Lancet,' (June and July, 1844,) on the detection of poisons generally in medico-legal cases, and on a new method for the detection of *arsenic*. He considers that a method laying claim to general applicability should fulfil the following conditions: "1. It must admit of detecting arsenic in every form in which this mineral can possibly exist. 2. It must not merely lead to the detection of arsenic, but also to that of the other metallic poisons. 3. It ought to preclude the possibility of confounding arsenic with other substances. 4. It must admit of detecting even very minute quantities of arsenic. 5. The method sought must enable us to obtain at least an approximate quantitative determination of the arsenic detected. 6. It must fulfil all these conditions by the most simple means." However desirable it may be to possess such a method as is here sketched out, the absolute necessity for it is not apparent. Arsenic may be most satisfactorily detected by processes which are not fitted for the detection of other metallic poisons. If each poison has its own particular process, and this is satisfactory so long as it is confined to its proper object, it is impossible to allow that the admissibility of chemical evidence in cases of arsenical poisoning, should be made to rest on the universal application of the same process, and with a like degree of certainty, to other poisons. Dr. Fresenius complains that those who have written innumerable essays upon this subject, have not always had "a very complete or distinct notion of the nature of their task; and they have therefore altered and improved methods *ad infinitum*, which in their very principle could never answer the design that they were intended to subserve."

Out of the many processes suggested for the detection of arsenic in mixtures containing organic matter, there are, according to Dr. Fresenius, only *four* which require to be mentioned. Arsenic may be separated, 1, as arseniate of lime; 2, as sulphuret; 3, from arsenuretted hydrogen; 4, by metallic copper. Of these methods, the second alone is recommended as fulfilling the conditions required, although it is obvious that the poison may be administered in the state of sulphuret, (yellow arsenic;) and therefore this, without some preliminary preparation, cannot fulfil the first condition required, i.e. of detecting arsenic in *every form* in which it can possibly exist. On the separation by lime nothing need be said, as it is now abandoned by toxicologists. Dr. Fresenius considers that the separation from arsenuretted hydrogen (Marsh's test) is absolutely inapplicable to the intended purpose, "because it does not admit of the separation of arsenic in every form in which this substance may exist." Further, it does not contribute to the detection of other metallic poisons; it contaminates the substance under investigation with zinc, which might itself have acted as the poisonous agent; it leads more easily than any other method to mistakes; and does not admit of any correct quantitative determination of the arsenic found. Marsh's test, it is allowed, permits the detection of minute quantities of arsenic in many cases in a very simple manner. Reinsch's process, i.e. the separation by copper, is just as little adapted to the purpose, although, like the preceding test, it may serve to detect very minute quantities of arsenic. "The defects of this method are that it does not admit of the detection of arsenic in every form in which the metal may exist; that it does not at all lead to the detection of other metallic poisons; and that moreover the substance under investigation becomes contaminated by copper. Its success is impeded or prevented by many substances, such as nitrates (?), mercurial and other metallic compounds; so that the advantage of detecting even minute quantities of the poison, can only be conditionally conceded to it. Finally, it does not allow of the quantitative determination of the arsenic present."

It appears to me impossible to assent to the validity of these objections, or to admit that we should be at all justified in entirely discarding the ingenious processes of Marsh and Reinsch, upon such grounds as are here adduced. These tests are fully equal to the separation of arsenic in all the forms in which it is most commonly found in practice; that they do not detect all other metallic poisons, or that their operation on arsenic is occasionally rendered obscure by the presence of other substances, are objections which amount to nothing in the hands of those who limit the application of these tests to the purposes for which they were originally designed; and it is doubtful whether they are more liable to lead to fallacy in skilful hands, than the process by conversion to sulphuret. Neither Marsh's test nor Reinsch's test will answer all the artificial conditions laid down as necessary for a universal method by Dr. Fresenius; but it is questionable how far it is just to measure the practical utility of these tests by such a standard as is here assumed.

Before converting the poison to the state of sulphuret, Dr. Fresenius considers the different plans which have been recommended for obtaining a clear solution, without loss of arsenic, from the organic mixture containing the poison. He gives the preference to the action of chlorine, as a decolorizing agent, and to the process of charring by sulphuric acid. As his method of employing chlorine for removing and destroying organic matter, and at the same time rendering the arsenic soluble, is novel, it is here briefly described with the analytical process.

The substances intended for examination are, if formed of solid and coherent lumps, reduced into small pieces, and under all circumstances carefully intermixed. They are then put into a porcelain basin, and drenched with an amount of pure concentrated hydrochloric acid, either equal or superior to the weight of the dry substances, and with as much water as will give the consistence of thin pap to the whole mass. The basin is then heated in the water-bath, and chlorate of potash, in portions of about half a drachm, is added to the mixture at intervals of about five minutes, and until the contents of the basin have assumed a bright yellow colour, perfectly homogeneous, and a thin liquid consistency. When this point is attained, about two drachms more of chlorate are added, and the basin removed from the water-bath. When cool, the contents are placed on a filter of linen or paper, and are allowed to run off: the residue is washed with hot water until the liquid which passes (which is also collected) is no longer acid. The whole is then concentrated in the water-bath, during which process it changes from a bright yellow to a brownish tint, and a saturated solution of sulphurous acid is added to this residue until the smell of sulphurous acid is clearly perceptible. The whole mixture is then heated for about an hour, until the excess of sulphurous acid is completely expelled. A current of washed sulphuretted hydrogen is now slowly transmitted through this liquid for about twelve hours. The glass containing the precipitated sulphuret is lightly covered, and kept at a temperature of about 86° until the smell of sulphuretted hydrogen has disappeared. The precipitate which, besides sulphuret of arsenic, may contain organic matter and other metallic sulphurets, is collected and washed on a filter, and afterwards dried with it in a porcelain basin heated by a water-bath. Fuming nitric acid is then added drop by drop, until the whole is moistened; it is then evaporated to dryness. Pure hydrated sulphuric acid previously heated is then added to the residue, so as to moisten it uniformly: the mass is again heated in a water-bath for the space of from two to three hours, and finally in a sand-bath, at a temperature of about 300°, until the charred mass begins to crumble. The residue is digested in water, filtered, mixed with hydrochloric acid, and the filtered liquid precipitated by sulphuretted hydrogen. The precipitate thus obtained is drenched with a solution of ammonia, and the ammoniacal fluid is evaporated in a balanced porcelain basin dried and weighed. The arsenic thus obtained as sulphuret may be quantitatively determined in the usual way. The residue, insoluble in ammonia, may be tested for lead, bismuth, copper, and mercury. The metallic arsenic is obtained from the sulphuret by heating it in a current of well-dried carbonic acid, with a mixture of carbonate of soda and cyanide of potassium as the reducing agent.

With the exception of the plan for removing the colour of the organic liquid containing arsenic, there does not appear to be any originality about this process. It is undoubtedly much more complex than some of the others for which it is recommended as a substitute; and unless it were conducted by one well versed in chemical manipulation, it might equally lead to error. The value of the sulphuretted hydrogen as a precipitant has been long known, and is fully appreciated by toxicologists; but this does not preclude the employment of other more ready and equally certain means for detecting arsenic. The process appears to be rather adapted for separating arsenic from its ores, than for detecting it as a poison in medico-legal cases. In practice we do not find the compounds of lead, bismuth, copper, and mercury, mixed with arsenic; and therefore it is useless always to have recourse to a process which invariably presupposes the admixture of these metals with the poison criminally administered. Even in cases of suspected compound poisoning, rare as they are, there is no difficulty in discovering the foreign metal mixed with arsenic by appropriate tests; and a court of law requires to know whether arsenic was present and was the cause of death, rather than whether it was mixed with traces of bismuth or lead, a fact, however interesting in a chemical, is wholly unimportant in a medico-legal view. It is doubtful whether by this process the absorbed arsenic could be detected in the soft parts of the body, with the same readiness as by the test of Marsh or Reinsch, and it is apparent that this is likely to become an important branch of medico-legal research. In short, if this process were to be admitted as the only certain and satisfactory method of detecting arsenic, it would restrict all medico-legal analyses of the poison to a few who are in the daily habit of exercising themselves in chemical manipulation. But the evidence given on numerous criminal trials shows that there are many practitioners who are able to demonstrate satisfactorily the presence of arsenic in mixed liquids, without having recourse to such an operose method as is here pointed out. Dr. Fresenius recommends cyanide of potassium and carbonate of soda as the reducing agent for the sulphuret. I have lately found that one of the best methods for reducing this compound of arsenic, is finely powdered metallic silver. This was recommended about a year since by Dr. A. Frampton for the reduction of corrosive sublimate,—it will be found equally serviceable in the reduction of the sesquisulphuret of arsenic.

In a paper read before the Sheffield Medical Society, and published in the Medical Gazette (May, 1844), Dr. E. Shearman states that the metallic ring, as shown in the reduction-test, can be produced by other metals besides *arsenic*. He also states, that the metallic arsenic may be known by its being deposited in rhomboidal crystals,—that a witness is justified in swearing positively to the presence of arsenic, only, "1, by producing the metal and showing its crystals; 2, by reducing it to the oxide and showing its crystals (octohedral); 3, from these crystals going through all the fluid tests; 4, reducing the sulphuret again to its metallic state, then to the oxide, and again going through the fluid tests." This is like demanding proof *ad infinitum;* for where, it may be asked, is the medical jurist to stop in his analysis? If he obtains the metal, it is said, "Antimony, bismuth, tin, zinc, lead, tellurium, cadmium, selenium, and potassium, sublime in a somewhat similar manner to arsenic, *and may be mistaken for it.*" Medical jurists have hitherto agreed on one point, namely, that the first five metals mentioned never yield, in a small reduction tube and at the ordinary heat of a spirit-lamp, any metallic sublimate whatever. With regard to the other four substances, cadmium is the only body that presents any objection to the reduction-test; and this is rather theoretical than practical. The cadmium-sublimate can never be mistaken for an arsenical sublimate by one who has been accustomed to experiment on the poison. It has a tin-like lustre, and generally a brown fringe of oxide, which, if not developed during the reduction, is readily brought out on re-heating the sublimed metallic crust. It is difficult to conceive under what circumstances tellurium, selenium, or potassium, can ever be sublimed in a reduction tube from any solid or liquid substance, handed to a medical jurist for analysis. There is no salt of potash that will yield potassium by heating it with flux in a spirit-flame; and if the metal were used in its pure state, no chemist could proceed to examine it as an arsenical compound without being made immediately aware of its nature. Admitting that the metallic arsenic is deposited in rhomboidal crystals, to be seen only "by a powerful microscope," there can be no better or more satisfactory evidence of its nature, than the production of white octohedral crystals, by reheating it, as suggested long since by Dr. Christison. Dr. Shearman states that metallic antimony sublimes into octohedral crystals like arsenious acid, only differing from this substance in being insoluble in water. In frequently repeating these experiments with a spirit-lamp and tube, such as are used in the analysis of arsenic, nothing of the kind was observed. A powerful microscope was not employed, because this is not needed in these investigations. If such a statement were borne out by observation, Reinsch's test, and the reduction-process for arsenic, would be completely set aside, and chemists would be thrown back upon the liquid tests for the poison. Many toxicologists have submitted Reinsch's test to numerous trials, but in no instance, so far as I am aware, have they ever procured a sublimate in white octohedral crystals from a deposit of antimony on copper. Dr. Shearman is entirely opposed to the opinion of Dr. Fresenius as to the value of the sulphuretted hydrogen test. Dr. Shearman says, "It should be *carefully remembered*, that the *sulphurets of antimony, tin, selenium,* cadmium, and tellurium have nearly the same yellow colour, and are deposited in the same manner as arsenic, and when reduced to the metallic state with black flux, they not only give an appearance so much like arsenic, that it requires a very practised eye (?) to distinguish each, if even that be possible; and tellurium and cadmium also exhale a garlic odour like arsenic. (?)" Dr. Fresenius actually stops at the production of the metal from a precipitate obtained by sulphuretted hydrogen;—he observes that "mirrors of wonderful purity are obtained *without the possibility of confounding them with those produced by any other substance.*" Thus Dr. Fresenius regards the medico-legal evidence complete, at the very point at which, according to Dr. Shearman, it becomes doubtful and unworthy of confidence. Toxicologists, it appears to me, will agree that in this respect Dr. Fresenius is correct; and that no practised eye can ever confound the metallic sublimate derived from sulphuret of arsenic or the white octohedral crystals obtained by reheating it,—with the metallic sublimate procured from any other substance precipitated by sulphuretted hydrogen.

Dr. Shearman also remarks, in respect to the alleged antidote for arsenic, "the moist hydrated peroxide of iron," that he has given it to dogs and rabbits after having made them swallow large doses of arsenious acid in powder and solution. On killing them within a short time and examining the bodies, he found in the stomachs, "minute patches of inflammation, but no arsenious acid could be detected by Reinsch's method," nor in any other way. This he considers is "a strong presumption, that the whole of the arsenic was reduced to its metallic state." If a large dose of poison had been given, and none found in the contents of the stomach, either free or mixed with the oxide of iron, it is clear that it must either have been rejected by vomiting, absorbed into the system, or that it must have passed off by the intestines. The fact of the animals having been speedily killed rendered the latter supposition improbable, therefore it is difficult to explain why arsenic was not discovered, if not in the viscera, at least in the soft organs. In experiments on this subject, one point has always been omitted, although it would throw considerable light on the action of the supposed antidote, namely, an analysis of the blood and soft parts of animals to which the oxide of iron has been given after they have been made to swallow large doses of arsenic. There is another way of testing the efficacy of this antidote, which has not been adopted, but which is worthy of a trial, by those who suppose that the oxide of iron is efficacious in cases of arsenical poisoning, namely, to mix powdered arsenious acid with the oxide of iron into a thin paste, and spread it upon a raw ulcerated or wounded surface. The antidote is said to act by preventing the absorption of the poison; if this were so, no evil should follow from the performance of such an experiment, however large the quantity of arsenic. On the contrary, Professor Barzellotti of Pisa found that a compound of this kind acted energetically as a poison on dogs, and the arsenite of copper, which is just as insoluble as arsenite of iron, would very speedily destroy life when thus locally applied. It is, however, impossible to understand how, in any case, the oxide of iron should act as Dr. Shearman suggests, by reducing arsenious acid to the metallic state!

In the year 1843, a great improvement was made in the chemical processes for arsenic by the discovery of *Hugo Reinsch*, that the metal was readily precipitated on *metallic copper*, even from the most complex organic liquids, by boiling them with muriatic acid and then introducing the copper. An account of this test was first published in the 'Journal für Praktische Chemie,' a translation of which will be found in the 'Annales d'Hygiène,' t. xxix, pp. 439-447, with additional observations thereon by M. Gaultier de Claubry, t. xxx, p. 159. A full description of the mode of applying this new and useful test will be found in the 'British and Foreign Medical Review' for July, 1843, (No. XXXI, p. 275). Dr. Christison has also published his observations on the test in the 'Edinburgh Journal of Medical Science,' for September, 1843. The only additional remark which may be made with respect to this test, is, that it will perhaps be found better to employ fine gauze, made of woven copper wire, than plates of copper as originally proposed by Reinsch. The surface of metal is much greater, and the gauze may, after the deposit, be easily rolled up into a small bulk, so as to yield a large quantity of arsenious acid. The copper-gauze which I have used for this experiment, contained 16,400 apertures to the square inch.

Many cases have come to trial during the last year in which parties have been charged with the criminal administration of arsenic. Among these some have presented circumstances interesting to the medical jurist. The case of the Queen *v.* Hunter has already been commented on (ante p. 534.) Another case was tried at the Liverpool Winter Assizes, 1843, (the Queen *v.* M'Cormick), in which the medical evidence was of some importance. A woman was charged with the murder of her child, aged five weeks, by administering to it arsenic. The quantity given was unknown, but the child did not die until twelve days afterwards. The symptoms do not appear to have been very severe or well-marked, indeed, so little so as to have led to some doubt of their cause in the mind of the medical witness. The chemical evidence was, however, very clear: arsenic was not only detected in some of the food given to the child, but also in a portion which had been vomited on its night-dress. It is stated that no arsenic was found in the stomach or duodenum; but the poison was detected in the contents of the intestines as well as in the substance of the brain, liver, lungs, and heart. In cross-examination, the witness was asked respecting the presence of arsenic as a normal constituent of the body. He is reported to have admitted that arsenic might be found in the body, although it had not been administered; but if found in the alimentary canal (free), then it must have been administered. He said that he himself had never found arsenic in the flesh of persons to whom it had not been administered; but he made the statement on the authority of others. He considered that it was not "normal" arsenic which had been found in this case; and that the ten thousandth part of a grain would give perceptible indications of the presence of the poison. The prisoner was convicted.

The mode of examination adopted at this trial clearly shows that barristers are well aware of Orfila's researches; but we have here an instance of the revival of an opinion which has now been for some time abandoned, even by Orfila himself—of the existence of arsenic as a natural constituent of the body. It had no influence on the result of this case; but it might have improperly led to the setting aside of the chemical evidence altogether, had not some free arsenic been found in the intestines.

One of the most important trials which has occurred for some years, was that of Mrs. Cochran or Gilmour, before the High Court of Justiciary at Edinburgh, January, 1844. She was charged with the murder of her husband by arsenic, but acquitted on a verdict of "*not proven*," apparently for want of clear evidence of administration. This trial is interesting from the fact, that the new methods of detecting arsenic were here, for the first time, brought prominently forward. The chemical evidence was complete, and such as left no doubt that the deceased must have died from the effects of the poison. A very full report of this trial has been published, to which I would refer the reader. Drs. Wylie, M'Kinlay, and Christison gave evidence on the occasion. Dr. Christison employed Reinsch's test, and by this means readily detected arsenic in the substance of the stomach and its contents, as well as in the substance of the liver. The same question, relative to the existence of normal arsenic, was put here as in the case of M'Cormick. In answer to it, Dr. Christison says, Arsenic " is not a constituent part of the human body, and is not formed in it. It was once alleged that it was, but that was disproved. The individual (Orfila) who first promulgated this theory only argues now, that small quantities are found in the bones; but in three several experiments before the Academy of Paris he was unable to show it. Arsenic could only have come into the liver by absorption." There can be no doubt that this expresses the truth. The elaborate researches of MM. Danger and Flandin show that there must have been some fallacy attending the original experiments of Orfila, since, in no instance, in operating on the largest quantities of animal matter, and employing the most delicate tests, could they detect the smallest trace of arsenic as a natural constituent of the human body. (See No. XXV of this Journal, p. 183.)

In concluding this account of arsenic, it is right to state, for the information of those engaged in medico-legal analyses, that there is still much sulphuric acid in general use which contains arsenic in large proportion. This is well known to be the acid obtained from pyrites. An impregnation of this kind seriously affects the use of the acid, both as an agent for carbonization and as a means of procuring hydrogen by Marsh's test; and no specimen should be employed for either purpose, until the absence of arsenic has been clearly established by analysis. The best means of detecting this body in sulphuric acid will be by the use of Reinsch's test, or by the action of a current of sulphuretted hydrogen gas on a portion of the acid, diluted and neutralized by potash. The presence of arsenic in sulphuric acid is the cause of the commercial muriatic acid being sometimes contaminated by it. Hence the necessity, in employing Reinsch's test, of being well assured that the muriatic acid used is free from arsenic. M. Scanlan states that the quantity of arsenic in one specimen of English sulphuric acid was so great that it could not be used to obtain hydrogen for the process of antogenous soldering. (Pharm. Journal, August, 1844.) This gentleman found that 2000 grain measures of the acid yielded 1·5 grain of sesquisulphuret of arsenic.

*Mercury. Albuminous Antidote.* Much discussion has arisen among toxicologists respecting the nature of the compound formed by albumen, when exhibited in cases of poisoning by corrosive sublimate. The great practical question is as to how far it is capable of disarming the poison of its virulence, and upon this most are agreed, namely, that it is a useful counteragent. Orfila has found that the compound may be given in large doses without danger, that it is soluble in a large excess of albumen, and then becomes poisonous, but less so than corrosive sublimate. The common practice in using albumen is to give only the white of egg, but, chemically speaking, the yelk, which is composed of the same principle, with a small quantity of oil, is just as efficacious.

With regard to the compound formed, Orfila's opinion was that the corrosive sublimate was reduced to the state of calomel by albumen, and thereby rendered inert. Lassaigne stated, from his experiments, that the albumen directly combined with the corrosive sublimate and formed an insoluble substance. A writer in the 'Dublin Journal of Medical Science' (May, 1844,) has lately called the attention of toxicologists to the experiments of Professor Rose which correspond in their results with those performed by himself. Prof. Rose considers the compound to consist of albumen united to the peroxide of mercury, and there is no doubt that a compound similar to, if not identical with it, may be at once formed by rubbing up fresh albumen with hydrated peroxide of mercury. The same may be procured by precipitating with albumen " a solution of pure pernitrate of mercury, as nearly neutral as possible." If added to the protonitrate of mercury, the protoxide is thrown down of a grayish-black colour.

In performing lately some experiments on the subject, I have found that the compound, produced directly by the admixture of albumen with the hydrated peroxide of mercury, possesses all the chemical properties of that produced by the action of albumen on corrosive sublimate. Thus it underwent similar changes when treated with chloride of tin, metallic copper, caustic potash and concentrated muriatic acid; but there was one difference, namely, that a small portion of corrosive sublimate was held combined with the precipitate formed in a solution of that poison by the addition of albumen. Albumen was added to a solution of corrosive sublimate, in sufficient quantity to produce the usual dense white precipitate, but not to redissolve it. The clear liquid was poured off, and the precipitate was afterwards thoroughly washed on a filter, until the washings gave no indication of the presence of corrosive sublimate. On adding potash to a portion of the precipitate, there was no apparent change, but on boiling a larger quantity of it in water, filtering and evaporating on a glass plate to crystallization, some minute white prismatic crystals were obtained, which were immediately turned scarlet on touching them with iodide of potassium. They were proved to be corrosive sublimate. The compound was then allowed to dry, when it formed a horny transparent mass. This readily dissolved in boiling concentrated muriatic acid, giving the usual deep purple colour formed by that acid with albumen. On diluting it with water, a precipitation of albumen took place, and the liquid gave an abundant metallic deposit on fine copper gauze. When this was dried and heated in a reduction tube, well-defined globules of mercury were obtained by sublimation. One fact appears to be obvious from this experiment, namely, that admitting the antidotal compound to consist of albumen and peroxide of mercury, it does nevertheless contain some undecomposed corrosive sublimate, not separable by mere washing with cold water, nor detectable by the addition of potash to a small quantity of it, but rendered demonstrable by long boiling in water and subsequent filtration and evaporation.

*Alleged poisoning by blue pill.* The account of an inquest on a person alleged to have died from the effects of *blue pill*, is reported in a contemporary journal (Medical Gazette, October, 1843.) It appears that the deceased, æt. 40, took some medicine prescribed for him by a practitioner. It consisted of *six grains* of blue pill and three of calomel. This was alleged to have produced salivation and a mercurial fever, of which the man died in about seven weeks. The salivation was probably owing to a remarkable idiosyncrasy, for a smaller dose than that here prescribed has been known to cause fatal salivation. But from the evidence, it was not improbable that the deceased had taken some quack pills which, had their composition been known, might have accounted for the severity of the symptoms. The jury returned a verdict of natural death, but called the remedy administered "an overdose of strong medicine!"

*Cancrum oris.* A case of cancrum oris in a child, mistaken for *mercurial poisoning*, has been communicated to the 'Medical Gazette' by Mr. Dunn of Norfolk-street. (Vol. xxxiii, p. 57.) Cases of this kind are of great importance, because they often involve practitioners in charges of malapraxis. An abstract is therefore given from Mr. Dunn's report. A girl, aged two years and a quarter, was brought to Mr. Dunn, on the 16th September, 1843. The child had an expression of heaviness about the eyes, the skin was hot, and the pulse quick. The mucous membrane of the mouth was in an unhealthy state, and the gums were spongy; there were blotches upon the body resembling the pustular form of scabies; the child was of a cachectic habit, from residing in an unhealthy locality and from defective nutrition. The following medicines were prescribed: A mixture of carbonate of magnesia and soda; three alterative powders, each containing pulv. rhei, gr. iv, sodæ exsicc. et hyd. c. cretâ (āā gr. ij) alt. noct. sum.; and some camphorated sulphur ointment to be applied to the blotches. One powder only was given; and when the child was seen two days afterwards, the eruption of measles was coming out, but not freely. She was then in a low drowsy state, and there was great prostration of the vital powers. Previously to the child's illness the gums had bled freely, their texture was now of a livid hue and spongy. At the junction of the gums with the lining membrane of the lower lip in front, there were a number of small yellow spots, resembling aphthæ, with a whitish exudation, the intervening mucous membrane being tumefied and red. Mel boracis was used, ten grains of the *compound jalap powder* were given, and a saline mixture with ammonia. Next day the aphthous spots presented irregular ulcerations, with ragged edges; these slowly extended, becoming of a dirty gray colour, and they were covered with a tenacious purulent exudation. The disease extended to the upper lip, right cheek and gums, and the breath was intolerably fetid. In spite of the application of the usual remedies, the disease gradually progressed in the dry form, the gums of the lower jaw were reduced to a black fetid pulp, and the child herself removed the whole of the teeth, one by one. The lower lip and chin became involved, and the first external eschar appeared on the chin, about a week after the appearance of the measles; the lower part of the face then became a black, soft, and homogeneous mass, having a gangrenous fetor. The child died fifteen days after the time at which she was first seen. An inquest was held, and it was alleged that ten grains of the hydr. c. cretâ had been given to the child twelve days before her death, instead of compound jalap powder. There was no proof of this, nor did it appear that the child had throughout her illness taken more than two grains of the hydr. c. cretâ, about a fortnight before her death. The medical evidence satisfactorily proved that there had been no improper treatment. The post-mortem appearances met with were as follows: "The right side of the face, half of the nose, and upper and lower lip were perfectly black. Upon carefully examining the inside of the mouth, there was observed great ulceration of the gums. The alveolar processes were denuded, and the teeth gone. Half of the tongue was black, and the inside of the cheek and fauces gangrenous, the whole exhibiting *the true "cancrum oris,"* or gangrene of the mouth. The stomach was perfectly healthy, and the small intestines diaphanous."

The fact that this was a case of cancrum oris appears to be established affirmatively, by the well-marked characters of the disease; and negatively, by the small quantity of mercury which had been taken by the child at a long period before the serious symptoms came on. This disease has not received so much attention from practitioners as its importance really deserves. One of the best descriptions of it has been published lately by Dr. Hunt in the 'Medico-Chirugical Transactions.' (Second Series, vol. viii.) According to this gentleman, it commences by small ulcers, either on the inside of the cheek or at the point of junction of the mucous membrane of the cheek and gums, or in the gums themselves, separating them from the teeth; they are very painful and tender, and accompanied by profuse salivation. The breath soon becomes tainted with an offensive smell, not unlike the mercurial fetor. If the disease be neglected, the ulceration goes on to destroy the gums, the teeth become loose and fall out, and the alveolar processes are laid bare. The brown ragged ulcer spreads rapidly on the inside of the cheek; the integuments over the spot corresponding to the ulcer become hard and swollen—at first white, and afterwards of a dull red colour, and shortly a black spot appears in the centre, which quickly spreads, and destroys more or less of the cheek. Should the child survive, there is much deformity, and it loses the power of opening its mouth, from the unyielding nature of the cicatrix; but more commonly, when the disease has gone to this extent, the child sinks and dies.

*Tests for corrosive sublimate.* It has been objected to the ingenious test proposed by Dr. Frampton for corrosive sublimate, either solid or in any state of admixture, (Medical Gazette, June, 1843,) that it will not answer in all cases, and that it is inferior in delicacy to tin and the chloride of that metal. Direct experiment, however, shows that these objections are more theoretical than practical. Metallic silver in a finely pulverulent state is easily procurable, and its efficacy as a test may be made evident by the most simple experiments. It has many advantages over the chloride of tin, and acts so perfectly in separating mercury from all solutions of corrosive sublimate, that there is no good reason why metallic tin should be substituted for it. Dr. Frampton, in carrying out his experiments on this subject, has discovered a fact of some importance in a medico-legal view, namely, that when the poison is in an extreme state of dilution, the mercury still admits of separation by boiling the liquid for some time with metallic silver. (Medical Gazette, Oct. 1843.) Dilution, it is well known, materially affects the action of all liquid tests; but in this case, except that the operation goes on more slowly, it does not appear to interfere with the action of silver in separating mercury. Dr. Frampton obtained distinct globules of mercury, by the use of silver, from a mixture containing *one tenth of a grain* of corrosive sublimate in *twenty-five ounces* of river water. As the boiling was continued for some time, there was a loss from evaporation, so that twenty-three ounces of fluid only were drained off; but, at the lowest estimate, there was a sensibility to one part of poison in 115,000 parts of water. A ring of mercury was also obtained in a case where one sixteenth of a grain of corrosive sublimate was mixed with twenty-seven ounces of water, in which case twenty-three and a half ounces were drawn off, thus proving a sensibility to one in 180,000 parts, and probably this is not the extreme limit of its power.

These experiments clearly show that copious dilution does not impair the action of the silver test—a very important fact where the contents of the stomach are very large: but it is to be remarked, that in both of the above cases, the test was brought into contact with the whole quantity of the poison used; this being in one instance one tenth, and in the other one sixteenth of a grain. There is no doubt that the test will detect a much smaller quantity of the poison than was here employed, and that the 100th of a grain may be easily discovered by it. This degree of delicacy is sufficient for all practical purposes. It has been further objected to the test, that in some cases the poison may be reduced to the state of metallic mercury by certain organic principles. Such an objection applies, however, no more to the silver than to the other tests, such as copper or tin and its chloride. The mercury must here be brought into a soluble state, by the well-known usual processes, before any test whatever can be made to act upon it.

*Conversion of calomel into corrosive sublimate by alkaline chlorides.* A case lately occurred in France, in which a medical practitioner was charged with the death of a child by the administration of a common dose of calomel with muriate of ammonia. It was stated by M. Mialhe, who gave evidence on this occasion, that, by contact with any chloride, such as common salt, calomel was converted to corrosive sublimate; and such a mixture was therefore highly dangerous. Experiments subsequently performed, showed that if this change did take place at all at common temperatures, or at the temperature of the stomach (98°), it was only to a trifling extent, and not likely to endanger life by the usual mode of exhibiting the medicine. The question is of some importance to medical practitioners; for, although it is not customary to give calomel in mixtures with alkaline chlorides, yet common salt is largely employed as an article of food, and the chlorides of sodium and potassium exist in the animal secretions. It is therefore proper to state here the results of some recent experiments on the subject by M. Larveque, especially since these tend to show that the statements of M. Mialhe are not strictly borne out by observation. An account of these experiments will be found in the 'London and Edinburgh Phil. Mag.' Sept. 1843. The principal facts merely are here selected. In one experiment a mixture was made of 45 grains of calomel, 90 grains of chloride of sodium, and 1875 grains of distilled water. The mixture was frequently shaken, but it was only after the lapse of a week that the supernatant clear liquid was at all discoloured by sulphuretted hydrogen gas. This was not, however, owing to the presence of any corrosive sublimate, for none could be separated by sulphuric ether. The effect of the gas was doubtless due to the presence of a minute portion of calomel held dissolved by the alkaline chloride. When nearly double the quantity of common salt was used with half the quantity of water, still no evidence of the production of any corrosive sublimate could be obtained. The chlorides of potassium, barium, calcium, and magnesium gave precisely similar results. When the mixtures were heated to 212°, then a portion of corrosive sublimate, easily separable by ether, was uniformly produced. Muriate of ammonia was found, even at common temperatures, to convert a portion of calomel to corrosive sublimate. This, however, is only likely to occur where the quantities of calomel and muriate are infinitely larger than it is probable they would ever be prescribed for medicinal purposes. Practically speaking, this conversion by common salt can never give rise to any dangerous consequences; because it is not found to take place at common temperatures, nor at the temperature of the body. The change produced by muriate of ammonia at common temperatures is so slight as to be of no importance.

*Lead.*—In November, 1843, an interesting trial took place at the assizes of the Puy de Dôme, in France, involving the rare question whether or not the death of a person had been caused by the criminal administration of a salt of lead. The whole of the proceedings are reported with much unnecessary prolixity (extending to 158 pages) in the 'Annales d'Hygiène' for January, 1844. The deceased died under suspicious circumstances; on examination of the body, there was nothing found indicative of the action of poison, while the stomach was ulcerated and in an otherwise diseased state. No salt of lead was found in the contents, but traces of the metal were discovered on incinerating the viscera. A question then arose, whether this metal was a natural constituent of the body, or the result of a portion which had been swallowed and had acted as a poison. The medical opinions were much divided. Orfila thought that it was very probable, if not certain, that the deceased had died from the effects of lead. There was so much doubt about the case, that, in an English court of law, it would probably have been speedily dismissed for want of clear medical proof of the cause of death. The details are not of sufficient general interest to justify quotation, but the medico-legal reader will find, in the controversy between MM. Dupasquier, Danger and Flandin on the one side, and M. Orfila on the other, that the art of conducting a medical prosecution and a *medical* defence is well understood in France.

*Carbonate of lead. Shot in bottles.* A case is related in the 'Annales d'Hygiène,' (April, 1844,) which shows that serious accidents may sometimes happen from the shot used in cleaning bottles being left, and afterwards becoming chemically acted on by the wine or liquid introduced. The practice of thus cleaning bottles is very common in England and also in France, and the small pellets often become fixed in the narrow part of the base of the bottle, and thus escape notice.

A person after having swallowed a few glasses of liqueur, suffered from the most violent colicky pains, and all the symptoms of irritant poisoning. Dr. Hanle, who was immediately called, having observed that the liquor remaining in the bottle was very turbid, poured it off for analysis, when he found, firmly wedged in at the bottom of the bottle, ten leaden pellets, which had become so completely transformed to carbonate of lead, that there was only a small nucleus of the metal left. So long as the liquor was clear, no accident had arisen from its use; but the symptoms of poisoning appeared immediately when the turbid portion, at the bottom of the bottle, containing the salt of lead either suspended or dissolved, was swallowed.

It is singular that the lead should have been found in this case in the state of insoluble carbonate; for, in general, the vegetable acids contained in wine (if we except the tartaric) form soluble salts of the metal. With acescent wines, such as those made in this country, which owe their acidity chiefly to citric acid, accidents of this kind are very liable to occur; but with good Spanish wines this is not so common. The acidity here is chiefly due to tartaric acid; and it is only slowly that tartrate of lead is formed, even when the quantity of shot left in the bottle is large.

*Action of water on lead.* A very important communication has been made on this subject by Dr. Christison to the Royal Society of Edinburgh, which has been since published in their 'Transactions' (Vol. xv, Part 2). A few years ago, the Doctor was led to examine the water introduced into a dwelling by a lead-pipe, from a distance of three quarters of a mile. It was remarked that the water fresh drawn from the pipe *was perfectly transparent at first,* but on exposure to air, it quickly presented a white film, afterwards ascertained to be carbonate of lead.

This water had been previously examined by Dr. Christison, and he had concluded that it contained salts enough to prevent corrosion of the lead, from the circumstance that several pieces of the fresh-cut metal retained their lustre when immersed in it for a period of fourteen days! "I did not," he observes, "at the time, advert to the difference between an experiment, in which some ounces of water were left at rest on a few square inches of lead, and one in which a column of water, only three quarters of an inch in diameter, flowed constantly over a surface of nearly 800 square feet." On analysis, this water was found to contain but a very small portion of saline matter (the 21,400th part), and the salts were of such a nature as to have the least protective influence. The remedy adopted, was to leave the spring water at complete repose in the pipe for a period of four months, so as to allow the carbonate to crystallize slowly and firmly on its interior. "This experiment was attended with complete success. The water was then found to flow without any impregnation of lead, and has done so ever since."

In another case, the water gave rise to the effect of slow poisoning by lead; and here again it was observed,—a fact hitherto not noticed by toxicologists,—that it was quite transparent when first drawn, and only acquired an incrustation of carbonate of lead after being exposed to air for some time. In this instance, the water contained a large proportion of saline matter (the 4460th part); but on analysis this chiefly consisted of chlorides,—the salts which have the least effect in preventing the action of water on lead; while the really preventive salts, commonly contained in terrestrial waters (sulphates and carbonates), were present only in the minutest traces. Polished lead was found to be tarnished by it in a few hours. The remedy adopted in this case was to keep the pipes constantly full of a solution containing a 27,000th of phosphate of soda. After the lapse of about three months, it was found that the water contained no traces of lead.

This last case establishes, that it is impossible to determine merely from the quantity of saline matter contained in water, whether it is liable to become impregnated with lead or not. The salts may be of a nature to have little or no *protective influence* in the proportion in which they exist.

Dr. Christison has found by his experiments, that the compound produced under these circumstances is not, as it is commonly supposed to be, a pure crystalline carbonate of lead, but that it is formed of two equivalents of carbonate of lead with one equivalent of hydrated oxide of lead. This compound is permanent in the air, and is only converted to neutral carbonate by suspending it in water and treating it with a stream of carbonic acid gas. It would appear from the analysis of Mulder, confirmed by that of Christison, that even the whitelead of commerce is not a pure carbonate, but a compound of four equivalents of carbonate to one equivalent of hydrate. The most simple method of preventing water from acquiring a poisonous impregnation by lead, available under all circumstances, is that of allowing it to remain for some months before use in the pipe or cistern. This gives time for a firm crystalline deposit of carbonate to attach itself to the surface of the metal, whereby all further action is prevented. It is not uncommon to find this deposit regarded by ignorant persons as the result of the hardness of the water;—it is scraped off and a fresh surface of metal exposed, so that in a case of this kind, water from a particular cistern may have been for a long time used with impunity,—and yet suddenly give rise to symptoms of lead-poisoning, probably to the surprise of the medical attendant and the parties affected.

The conclusions of Dr. Christison are of such importance in a medical view, that we here subjoin them. 1. Lead pipes ought not to be used for the purpose of conveying water, at least where the distance is considerable, without a careful chemical examination of the water to be transmitted. 2. The risk of a dangerous impregnation of lead is greatest in the instance of the *purest* waters. 3. Water which tarnishes polished lead when left at rest upon it in a glass vessel for a few hours, cannot be safely transmitted through lead-pipes without certain precautions. Conversely, it is probable, though not yet proved, that if polished lead remain untarnished, or nearly so, for twenty-four hours in a glass of water, the water may be safely conducted through lead-pipes. 4. Water which contains less than about an

8000th of salts in solution, cannot be safely conducted in lead-pipes, without certain precautions. 5. Even this proportion will prove insufficient to prevent corrosion, unless a considerable part of the saline matter consist of carbonates and sulphates, especially the former. 6. So large a proportion as a 4000th, probably even a considerably larger proportion, will be insufficient, if the salts in solution be in a great measure muriates. 7. It is right to add, that in all cases, even though the composition of the water seems to bring it within the conditions of safety now stated, a chemical examination should be made of it after it has been running for a few days through the pipes. For it is not improbable that other circumstances, besides those hitherto ascertained, may regulate the preventive influence of the neutral salts. (It may be here suggested whether organic matter in water, which has a strong tendency to combine with oxide of lead, may not have some influence.) 8. When the water is judged to be of a kind which is likely to attack lead pipes, or when it actually flows through them, impregnated with lead, a remedy may be found either in leaving the pipes full of the water and at rest for three or four months, or by substituting temporarily for the water a weak solution of phosphate of soda, in the proportion of about a 25,000th part.

I have found that sulphate of lime, when it forms about the proportion of a 5000th part, also acts as a good preservative. This salt, as plaster of Paris, is easily procurable in most localities.

A few years since, Dr. Clark of Aberdeen, suggested a very ingenious chemical process for depriving common water of two thirds of its solid saline matter, thus rendering it more soft and better adapted for many purposes to which it cannot now be applied. Two thirds of the saline matter, contained in the Thames river-water, consists of carbonate of lime held dissolved by carbonic acid; the remaining third is chiefly composed of sulphate of lime. Dr. Clark proposed to add caustic lime to water, in order to remove the free carbonic acid, and thereby to precipitate it, and the carbonate of lime held dissolved by it, entirely. The experiment perfectly succeeds when a few ounces of lime-water are added to a gallon of river-water;—any surplus lime is got rid of by exposure to air. When the precipitation has occurred, (occasionally not for some hours, or even a day,) the clear water may be poured off, and it will be found much softer. Having produced a quantity of this water, I was desirous of seeing how far, by the loss of so much saline matter, it would now be affected by contact with lead. Many experiments were performed on a small scale, and it was found that the preservative properties of the water were not at all diminished by the separation of the carbonate of lime. This seems to show that the sulphate of lime (the residuary salt) is mainly concerned in counteracting this chemical action between water and lead.

Mr. Scanlan has lately found that recently distilled water, condensed in a leaden pipe, holds dissolved a quantity of carbonate of lead, being turned brown by sulphuretted hydrogen. This is important in analysis, whether chemical or medico-legal. (Pharm. Journal, August, 1844.)

The most convenient plan, in Dr. Christison's opinion, for detecting lead in water, a duty which may occasionally fall on a general practitioner, is,—1. To examine what separates on exposure to the air, by dissolving it in warm acetic acid, and testing the solution with sulphuretted hydrogen, iodide of potassium, and bichromate of potash. 2. If this process fail,—To concentrate the water to an eighth part, and again test any insoluble matter which separates; and lastly, failing this procedure also, to evaporate the water to dryness, subject the residue along with charcoal to a red heat, act on what remains with warm diluted nitric acid, and test the solution when filtered and neutralized by an alkali. It may admit of question, whether in the event of lead being indicated in the last way only, the very minute quantity which would then be present, can prove detrimental.

We have assigned this space to one of the most valuable monographs on lead-poisoning which has appeared within the last few years. It is a subject of daily interest to the medical practitioner; and there are few cases in which chemistry has been brought to bear on medical police with more satisfactory results than in this. The cause of the mischief and the remedy are clearly pointed out. There is no doubt, that the dangerous effects produced by the contact of water with lead was well known to the Romans,—that this was the real cause of their abandoning its use, and, in the absence of iron,—resorting to those expensive structures of masonry which are now seen stretching in gigantic piles, and to enormous distances, over the Campagna di Roma. One of their leaden pipes has been found, and is now preserved in the Museum of Arles, with the name of the Roman plumber at every juncture. It is much to be regretted that there is even in our own day, among architects and builders, a degree of ignorance on this subject as great as that which existed two thousand years ago, and that notwithstanding the discoveries of modern science, serious accidents are frequently occurring in families from the want of the most simple precautions in the use of this metal.

*Colica pictonum.* In a paper read before the Academy of Sciences in November 1843, M. Ruolz proposed that the use of white-lead as a pigment should be abandoned, and that the white oxide of antimony should be substituted for it. Subsequently M. Rousseau suggested a process for the economical manufacture of the white oxide of antimony. It is not improbable that such a change might be attended with the benefit proposed—of extirpating colica pictonum and those disorders which affect individuals employed in white-lead works; but it is questionable how far the finely-divided oxide of antimony could be received into the system with impunity. Further, in respect to the arts, no substance has yet been found which has possessed, as a pigment, the degree of opacity which is known to belong to the carbonate of lead.

*Copper.* In July, 1843, a communication was made to the Academy of Sciences by MM. Danger and Flandin, on a new process for detecting copper, by the incineration of organic matter in cases of poisoning. By this means they have been enabled to detect the metal when it formed only the 100,000th part of the organic compound. (Annales d'Hygiène, t. xxx, p. 449.) The organic matter is simply dried and carbonized by heating it in a porcelain capsule, with one third of its weight of strong sulphuric acid. After being heated to dull redness, the charcoal is reduced to powder, drenched with sulphuric acid, again heated, but not to dryness, and then digested in water. By this means a clear solution is obtained, in which sulphate of copper is easily detected by the usual tests. The same process is applicable, according to the authors, to the detection of the compounds of lead, silver, bismuth, tin and gold, with the exception that, in the case of lead, muriatic, and of gold and tin, nitro-muriatic should be substituted for sulphuric acid, in acting upon the incinerated ashes. By direct analysis and physiological experiments, the authors have arrived at the conclusion, that neither lead nor copper forms any constituent part of the healthy animal body. In animals to which they had exhibited a salt of copper in divided doses, they found that the metal was not excreted with the urine, as in the case of arsenic and antimony; but with the bronchial secretion, in which they detected it by analysis. Mercury appears to take the same course in its exit from the body, since it has been found in the saliva of persons who have been taking mercurial pills. The compounds of silver and gold were observed to pass off both by the pulmonary and urinary excretions; but while the chloride of gold passed more readily by the kidneys than the lungs, it was exactly the reverse with the chloride of silver. After death from poisoning by a salt of copper, that portion of the metal which has been absorbed can be detected only in the intestines and in the liver; and about two ounces of this last-mentioned viscus were found to be sufficient for obtaining satisfactory medico-legal proof of the presence of the poison.

The question whether copper and lead constitute part of the healthy animal organs, in cases where neither of these substances can have been taken in a poisonous form, is a point which does not appear to be decidedly cleared up; although the balance of opinion is in favour of the results obtained by MM. Danger and Flandin, namely, that they neither enter into the composition of the body nor of the food of man; and that where they are said to have been detected, their presence must be ascribed to their adventitious introduction during the analysis. The question is of some interest in toxicology, for it has been already raised and thrown out as an objection to medical evidence in a late trial, already adverted to. (Ante, p. 538.)

In August, 1843, M. Barse communicated to the Academy the results of some analyses made on the bodies of two subjects taken from the hospitals of Paris. They had died from ordinary disease. M. Barse states that he detected copper and lead in both subjects. The copper was obtained in the metallic state and identified by all its characters; the lead was not obtained as a metal, but its presence was indicated by the usual tests. These metals may be detected in the liver, according to M. Barse, 1, by Orfila's process of carbonization; 2, by simple carbonization, incineration of the ash, and afterwards digesting it in nitro-muriatic acid; 3, in carbonizing by sulphuric acid and *incinerating the charcoal,* for the mere carbonizing action of sulphuric acid will not of itself suffice to allow of the detection of these metals.

In September, 1843, M. Rossignon of Lyons addressed a note to the Academy of Sciences on copper, as it exists in the organic tissues of many vegetables and animals. M. Rossignon states that he detected copper in all his experiments on the human body: he found it in the blood and muscular fibre of man, in the tissues of many domestic animals (the dog), and in the common vegetables used as food. The gelatin used as soup at the hospital of St. Louis yielded per cent. 0·03 of pure copper. Common sorrel gave 2 per cent. of neutral oxalate of copper; chocolate from 0·07 to 0·5 per cent. The bread generally used in Paris gave, in 1000 parts of incinerated residue, from 0·05 to 0·08 of copper (fraudulently introduced as sulphate?) Coffee, chicory, madder, and sugar yielded traces of the metal, in the latter case mixed with lead. Barley-sugar contains copper; and in the sugar of starch it forms 4 per cent. by weight, of the carbonized residue! M. Rossignon further states, that by calcining the substances in close vessels, he was enabled to detect appreciable traces of the metal in human semen, in the excrement of the fowl, in the egg, and in the eye of the ox!

It appears difficult to reconcile these results with those obtained by MM. Danger and Flandin and others. It is intimated that the failure of these experimentalists in detecting copper was owing to their not having incinerated the carbon derived from the action of sulphuric acid on organic matter; but this does not sufficiently account for the difference, since, by pursuing the same process with the pulmonary exhalations of animals poisoned with its salts, they detected the metal readily, although here it was only found in traces. Besides, when we consider the very positive manner in which it was for a long time stated that arsenic was a normal constituent of the human body, by a higher authority than either M. Barse or M. Rossignon, and that this statement has been since entirely disproved before a committee of the Academy, we may well hesitate to assent to the assertion that copper is a natural constituent of the body. These chemical mistakes are very liable to occur in researches conducted by individuals; and it is often only by the presence of several engaged in watching the process, that the real source of fallacy is detected. *Quod volumus facilè credimus.*

*Verdigris.* One case of poisoning by this substance is related in the 'Journal des Conn. Med. Chir.,' December, 1843. It is reported in the 'Edinburgh Medical and Surgical Journal' for July, 1844. A woman, ætat. 28, swallowed a large dose of verdigris. She was soon afterwards seized with great anxiety, vomiting, acute pains, and swelling of the abdomen, sensation of burning heat in the throat, coldness, and severe cramp in the extremities, a labouring pulse, swelling of the face, with the eyes sparkling. An emetic brought away some half-digested food, without any traces of poison. The next morning there was painful deglutition, swelling of the throat, the abdomen tympanitic and painful on the least pressure, the countenance heavy, the face flushed, and the pulse oppressed. About two pounds of a distinctly-greenish fluid, with some blood were ejected. The symptoms became aggravated; the face and eyelids swollen and red, the eyes prominent, the abdomen flattened but sensible, the rectum so irritable and painful that enemata could not be administered. On the second day there was a tendency to coma, the face was pale, the lips swollen, the gums ulcerated, and there was an abundant discharge of viscid saliva. A copious stool was passed—the first since the poisoning; and acetate of copper was detected in it in pretty large quantity. There were several spasmodic fits. On the third day some viscid glairy matter, of a greenish colour and tinged with blood, was vomited, and the spasms continued. On the fourth day epistaxis with general cramps came on, and the urine and fæces were suppressed. There was coldness of the surface, with convulsions. After the lapse of about a week she still had vomitings of greenish glairy matters, with uneasiness in the abdomen; but from this date she gradually recovered.

This case is interesting from the course of the symptoms being accurately noted; and it is worthy of remark, that icterus, which some have regarded as a symptom of cupreous poisoning, was at no time present. It is unfortunate that the quantity swallowed was not known.

*Subchloride of copper.* Among the very few cases of poisoning reported in Henke's Zeitschrift der S. A. for 1843-4, is the following. (No. i, 1844.) A boy between two and three years of age swallowed a part of a small cake of green water-colour, such as is sold in the colour-boxes for children. Very soon afterwards he was attacked with vomiting and coldness of the extremities. Notwithstanding the exhibition of an antimonial emetic, the symptoms continued to become aggravated, and the child died. On opening the body, there was nothing to indicate specially the action of an irritant poison, except a slight congestion in the cerebral vessels. The child, it appears, had swallowed about a scruple of the green colour, which, on analysis, was proved to be the common subchloride of copper. It was remarkable that there was not the least sign of irritation or inflammation in the alimentary canal. Death was ascribed by the examiners to the exhaustion resulting from violent vomiting, and the congestion of blood in the brain thereby produced.

This case, the details of which are rather imperfectly given, shows that the subchloride of copper is a very active poison, and that it may cause death without leaving any signs of irritation in the alimentary canal. It is to be remembered that it is this compound of copper which is often formed in culinary utensils, and which thereby gives rise to accidents when any food containing salt has been prepared in the vessel without proper precautions.

*Arsenite of copper. Scheele's green.* The dangerous practice of using this powerful poison to give a green colour to confectionary is very prevalent, and accidents are continually arising from this cause. An instance has just been communicated to me, of recent occurrence, in which three lives have nearly been sacrificed, at a school near Manchester, owing to the parties having eaten some ornamented confectionary, which owed its green colour to arsenite of copper. They suffered from violent vomiting, severe pains in the stomach and bowels, and spasms in the extremities. Three animals which ate of the vomited matters were attacked by similar symptoms. It is unfortunate that in this country there is no medical police established by law to restrict the free sale and use of this and other poisons. In this respect the English is widely distinguished from the continental practice. In France and Germany the lives of individuals are closely protected against those accidents which are liable to occur through the ignorance or criminal neglect of others. Here poison is allowed to be sold like sugar or starch; and every child is assumed by the law to be capable of protecting himself! If death ensue from such a cause, we find that a coroner's inquisition and a trial for manslaughter take place, to investigate an event which, under simple medical-police regulations, would not have occurred. More than a hundred lives are yearly sacrificed in England and Wales by the unrestricted manner in which arsenic is sold. The sale of alcohol is rigorously confined by fiscal regulations; and it is impossible to say why some strong restrictions should not be placed on the sale of poisons that can seldom be required by the public for any innocent or lawful purpose. If the sale were prohibited by a penalty, except under the order of a regular medical practitioner, it is quite certain that many lives would yearly be saved, and the painful proceedings connected with these criminal trials would be spared to the country. To those who are inclined to adopt the "*argumentum ad crumenam*" it may be observed, that the law-charges incurred for such inquisitions and trials, would more than pay for the establishment of a national board of medical police.

Arsenite of copper is not the only poison which gives a green colour to confectionary. Chromate of lead, mixed with indigo has also been employed for the purpose. It has been said that there is danger in the use of these compounds for tinting paper, should the paper be subsequently used for wrapping up articles of food. The reader will find in the Annales d'Hygiène (1843, p. 358,) a full account of the composition of these mineral colours, with the police regulations adopted in France on the subject. The green and bright blue papers are condemned as the most dangerous; but, unless the colour be only roughly adherent to the surface, it is not probable that an article of food inclosed in the paper would acquire a poisonous impregnation.

*Electrotyped copper utensils.* Mr. Warington has lately shown that copper vessels, saucepans, taps, and other articles, which have been covered with a surface of silver by the electrotype process, are liable to be acted on by weak acids, such as lemon-juice and vinegar, when such acids are allowed to remain in contact with them for a short time. It appears that the metallic silver with which they are covered is porous, like a sponge—a fact made evident on slight examination—and in this way the acid liquid permeates the silver, and reaches the surface of the copper. A kind of galvanic circuit is thereby established, which increases the chemical action; so that such vessels, while giving apparent security in their use, are actually rendered more dangerous. The presence of copper in acid liquids kept in the electrotyped vessels, was clearly proved by the usual tests for that metal. The same effects might occur where the liquid contained common salt dissolved.

*Antimony.* It would appear from the observations of the late Mr. Goodlad (Provincial Journal) of Manchester, and Mr. Noble, that tartarized antimony, even in small doses, is liable to act as a poison on the young. Mr. Wilton records four cases in which prostration and collapse followed the administration of ordinary doses of tartar-emetic to young children. Two of them were fatal. It should therefore be administered with great caution. A case, showing the importance of this remark in a medico-legal view, will be found in the Medical Gazette, vol. xvi, p. 520.

*Bichromate of potash.* Well-observed cases of poisoning by this compound, which is now extensively used in the arts, are rare; and, therefore, the details of the following case, communicated to the 'Medical Gazette,' (vol. xxxiii, p. 734,) by Mr. Wilson of Leeds, are of great practical interest. A man, ætat. 64, was found dead in his bed, twelve hours after he had gone to rest. He had been heard to snore loudly during the night, but this had occasioned no alarm to his relatives. When discovered he was lying on his left side, his lower extremities being a little drawn up to his body: his countenance was pale, placid, and composed; eyes and mouth closed, pupils dilated, no discharge from any of the outlets of the body, no marks of vomiting or diarrhœa, nor any stain upon his hands or person, or upon the bed-linen or furniture. The surface was moderately warm. Some dye-stuff, in the form of a black powder, was found in his pocket. On inspection, the brain and its membranes were healthy and natural; there was neither congestion nor effusion in any part. The thoracic viscera were equally healthy, as well as those of the abdomen, with the exception of the liver, which contained several hydatids. A pint of a turbid inky-looking fluid was found in the stomach. The mucous membrane was red and very vascular, particularly at the union of the cardiac extremity with the œsophagus; this was ascribed to the known intemperate habits of the deceased. In the absence of any obvious cause for death, poison was suspected, and on analysing the contents of the stomach, they were found to contain bichromate of potash; and the dye powder taken from the man's pocket consisted of that salt mixed with cream of tartar and sand.

It is remarkable that in this case there was neither vomiting nor purging. The salt does not appear to have operated so much by its irritant properties, as by its indirect effect on the nervous system. This, however, is by no means an unusual occurrence, even with irritants, far more powerful than the bichromate of potash.

*Antidotal treatment in cases of poisoning.* This subject has been lately examined by M. Bouchardat, and the conclusions at which he has arrived for the different irritant poisons are as follows: For *corrosive sublimate*, he recommends a mixture of zinc with iron filings, or iron reduced from the oxide by hydrogen, the moist per-(proto-?) sulphuret of iron. The same may be employed in cases of poisoning by copper and lead. In arsenical poisoning, the moist hydrated peroxide of iron, and the moist per-sulphuret may be used. This last preparation, it is alleged, decomposes the compounds of all of these poisons, and should, therefore, be exhibited when the nature of the irritant is unknown. The heavy metallic powders may be given in an electuary; the fauces should be tickled to promote vomiting. It is said to have been found that 100 parts of the powder of iron or zinc was a complete counteragent to fifteen parts of the acetate of copper; but it required a much larger quantity of the moist magma of the per-sulphuret of iron, to produce the same effect. (See Ed. Med. and Surg. Journal, April, 1844.)

An ingenious paper has lately been communicated by Sir George Lefevre to the 'Lancet,' (June, 1844,) in which will be found a summary of the common antidotes employed in cases of poisoning, with some excellent remarks on medical police, in reference to the sale and use of poisons. In a case of poisoning by *oxalic acid*, where the usual antidote is not at hand, Sir George recommends the administration of old mortar, finely powdered, or even the plaster scraped from a wall. In respect to other poisons, the only novelty of treatment which appears is in reference to *prussic acid*, where he states, that "a solution of sulphate of iron is found to be the best antidote," and even "*ink*" may be administered if the salt of iron should not be at hand.

There must surely be some mistake in reference to the use of sulphate of iron under such circumstances. The sulphate of iron cannot possibly act as a chemical antidote, for in solution it mixes with prussic acid in all proportions, without any chemical change whatever, a fact easily demonstrated by experiment. So, again, ink could have no more effect on the body than so much water, unless, as it sometimes happens, it should contain corrosive sublimate, (put into it for the purpose of preventing mould,) when the effect would be the reverse of favorable. Indeed it is difficult to conceive on what principle, except on the wrong supposition that Prussian blue might be formed by the admixture, such substances could be recommended as antidotes in cases of poisoning by prussic acid.

*New antidote to prussic acid.* Although neither sulphate of iron nor ink can be considered to possess the least antidotal power in cases of poisoning by prussic acid, yet there is one form in which iron may be used with some hope of success. The production of Prussian blue by an admixture of the protoxide and peroxide of iron, precipitated from the green sulphate by caustic potash, has been long known as an admirable test for prussic acid. The application of these mixed oxides, as an antidote in poisoning by prussic acid, has been lately proposed by Messrs. T. and H. Smith of Edinburgh; and it may be said of this antidote that, whenever it can be administered sufficiently early, there is a very reasonable prospect of its success; for, by mere contact, it speedily converts this formidable poison into the insoluble inert compound, Prussian blue. These gentlemen have not, so far as I know, yet published any detailed account of their experiments; but it is stated, that when the acid with the antidote was given to dogs, the animals lived, while the same dose of acid without the antidote, proved fatal. The method by which Messrs. Smith prepare the antidote is not announced; but I have found that it may be prepared by precipitating, in a closed vessel, a strong solution of the common green sulphate of iron by caustic potash, and washing the oxide repeatedly with water recently boiled, until the alkali and the surplus sulphate are removed.

The following experiment was performed, in order to test the efficacy of this antidote in a chemical view. Four drachms of prussic acid, containing 1 per cent. of anhydrous acid—a dose sufficient to destroy an adult in a very short period of time—were agitated with the mixed oxides of iron, precipitated in the manner described, from a solution of one ounce of crystallized green sulphate of iron. That

Prussian blue was almost instantly formed was evident on testing the mixture. It was allowed to remain an hour digesting; and then distilled at a very gentle heat. The clear liquid obtained by this distillation, was tested both by nitrate of silver and sulphate of iron. These tests had given abundant evidence of prussic acid in a few drops of the poisonous liquid, before the oxides of iron had been added; but now, not a trace of the poison could be discovered, neither by the odour nor by the action of nitrate of silver, which will detect the 1500th part of a grain of anhydrous acid; and for this experiment, as much as one fourth of the distilled liquid was used. No Prussian blue could be obtained, by the use of the sulphate of iron in the usual way, in operating upon another fourth of the distilled liquid; although this test is adequate to the detection of the fiftieth part of a grain of anhydrous acid, in a large quantity of water. It is therefore evident that the views of the Messrs. Smith are strongly confirmed by the results of these experiments; and now two questions will probably arise in the mind of the reader, respecting this antidote: 1. Whether, considering the great rapidity with which this powerful poison operates, any antidote whatever can be of the least service? 2. Admitting that the antidote is administered, is the conversion of the poison to the state of Prussian blue, so complete and so rapid as to counteract its effects?

With regard to the *first* question, it may be remarked, that if in only one case in a hundred, there is sufficient time for its administration, a life may be saved; and much credit is therefore due to the Messrs. Smith for the ingenious suggestion of such a remedy. It is true that the symptoms from a powerful dose of prussic acid come on commonly in about ten or twelve seconds; but so long as life continues, there may be a hope of recovery by neutralizing the effects of the poison; and the recent case of Mrs. Belany, who lived twenty minutes, and respecting the death of whom a trial has recently taken place in this metropolis, shows that instances do occur in which there may be time for antidotal treatment. With respect to the *second* question, the answer to this must depend in a great measure on the results of experiments. No inference could be fairly drawn from the administration to an animal of the oxides of iron already mixed with prussic acid; although this was the way in which the alleged iron-antidote, in cases of arsenical poisoning, was, in the first instance, most improperly tested. Such an experiment may prove, it is true, the inertness of the compound formed; but it is quite unfitted to illustrate the antidotal powers of the substance used; for a human being is never likely to swallow, as this experiment assumes, the poison and antidote together. In the case of prussic acid, more than in that of any other poison, it is necessary that the alleged antidote should produce its effects when administered a *few seconds or minutes after the acid has been taken.* On adding a small quantity of the mixed oxides to about one drachm of prussic acid, agitating and rapidly filtering, I found the poison still existing in pretty large quantity in the filtered liquid. I have reason to believe, however, that the chemical change would have been expedited by the use of a larger quantity of the mixed oxides; and as they are inert, there is no objection to their being given in very large doses. At any rate, it must, I think, be admitted, that, both in a chemical and pathological point of view, no remedy for prussic acid has hitherto been proposed, with a greater prospect of success than this possesses. If it should fail, its failure cannot be ascribed so much to any defect in its chemical operation as to the extraordinary rapidity with which the poison acts upon the system. The mixed oxides of iron may be prepared from the sulphate, and kept in a closely-stoppered bottle, filled with water previously deprived of air by boiling. Protoxide of iron may be speedily obtained in large quantity by agitating iron filings with a solution of sulphurous acid for a few minutes; filtering, adding caustic potash, and washing the precipitate with well-boiled water. It is important to state, that *peroxide of iron,* (the substance used in cases of arsenical poisoning,) cannot, in this case, be substituted for the mixed oxides; although it may serve to mix with the protoxide prepared in the way just described. The peroxide of iron alone will not form Prussian blue under any circumstances with prussic acid. In poisoning by the essential oil of bitter almonds, or by cyanide of potassium, the mixed oxides of iron are equally applicable; and in one case related in this Report, of poisoning by the oil of bitter almonds, the antidote might, had it been then known, have been used, and have accelerated recovery. It is possible, indeed, that it may become of more frequent use as an antidote to the oil, than to prussic acid. With respect to cyanide of potassium, we may at once administer a solution of green sulphate of iron to the patient, as the oxides are immediately precipitated by contact with the salt, and Prussian blue is formed. In the short account which I have seen, the Messrs. Smith appear to have confined their observations to the effects of the oxide on prussic acid.*

*Animal irritants.* In a late number of the 'Edinburgh Medical and Surgical Journal,' (July, 1844,) it is observed, in reference to the poisonous properties of the *flesh of diseased animals* used as food, that "in America there are certain regions, extending for many miles in length and some miles in breadth, on the herbage of which, if an animal feeds, its milk and flesh acquire poisonous properties, yet itself enjoys tolerable health. The disease which the use of the flesh or milk of the animals fed on these districts produces, is known over all America by the name of the milk-sickness, or 'trembles.' All the infected spots occur west of the Alleghanies; and it is well known, that of the early emigrants whole communities, on account of the prevalence of this malady in a particular locality, which is generally distinctly circumscribed, were often compelled to seek another; and even at this day, those who venture within the boundaries of an infected district, are constrained, as a condition of their residence, to abstain from the flesh of the cattle living within the same limits, as well as from the milk and its preparations. It appears from the late report of Drs. Hosack, Post, and Chilton on this subject, that in some of these infected districts, the inhabitants, with a recklessness of human life which seems incredible, carry the butter and cheese which they themselves dare not eat to the markets of the towns west of the Alleghanies, and that thus there are frequently produced symptoms of poisoning and even death, for which the medical attendant cannot account, or he is induced to consider as some new or anomalous form of disease. From the same report we learn that the cattle from these districts are sent in great droves over the mountains, but, in order to deceive the buyers as to the place whence they come, they bring them to New York by a southern route, and style them 'southern cattle.' The flesh of these animals produces, in those who make use of it, symptoms of aggravated cholera morbus. The viscera of the animals are often found diseased, and the livers almost invariably so."

Owing to the symptoms of poisoning which have followed the use of such beef, butter, and cheese, the American government caused a medical inquiry to be instituted into the matter; and it is probable that they will adopt the recommendation of the reporters, i.e. prohibit its sale. In the event of this occurring, it has been suggested as not improbable, that this poisoned food may find its way into England, and, from its cheapness, be diffused among the poor. It would therefore be advisable, that practitioners should be on their guard, and note any suspicious circumstances that may arise. As we are without a system of medical police in this country, it is not likely that government will have it in its power to prohibit the sale of such food, until many cases of the serious effects produced by it, have occurred.

There is another more common article of food, namely, *bread*, upon which some observations have been lately made by toxicologists. In the 'Annales d'Hygiène,' 1843, pp. 35 and 347, will be found communications on this subject from MM. Guérard, Chevallier, and Gaultier de Claubry. The changes which take place in the decomposition of flour and bread, and the production of various kinds of *mouldiness*, are here investigated, together with the effects of such bread upon the animal system. It would appear that in some parts of France the peasantry manifest no repugnance to the eating of *mouldy bread*; and that in many instances the practice appears to be attended with no ill effects. The nature of the mould produced, however, is subject to great variation, and it is not improbable, as M.

* It will of course be understood, that cold affusion with stimulants should be at the same time resorted to; and if the power of swallowing is lost, the antidote may be introduced by a stomach-pump.

Chevallier suggests, that in some cases a poisonous principle is actually developed. In two instances of children, who had partaken of mouldy rye-bread, symptoms resembling those of irritant poisoning, supervened. The countenance was red and swollen, the tongue dry, the pulse quick, there were violent colics, with pain in the head and intense thirst. Vomiting and purging supervened with a state of collapse, but the children eventually recovered. These symptoms were ascribed to the production of "*mucor mucedo*" in the bread. In 1829, alarming effects having followed from the use of a certain kind of bread in Paris, M. Burruel was called upon to determine whether or not any irritant poison had become accidentally intermixed with it. The bread was simply in a mouldy state; there was no trace of poison. It is unnecessary to enter further into this subject; the facts adduced, together with experiments performed on animals, show that bread, in a state of mouldiness, may not only produce symptoms of poisoning, but actually cause death; and as it is impossible to distinguish the noxious from the innoxious kind of mould, the use of all bread in such a condition should be avoided.

Even fresh bread may occasionally seriously affect the body. The brown bread of London has been known to produce vertigo, lethargy, and other unpleasant symptoms, indicative of an affection of the brain and nervous system. This has been ascribed, with some probability, to the "*lolium temulentum*" becoming accidentally mixed with the corn. Rye-bread is not much used in this country, but the presence of the ergot might here, in some cases, account for the symptoms which have been observed.

*Sulphate of potash.* The question whether this is to be regarded as a poisonous salt, of an irritant nature, has been much debated within the last year among members of the profession, owing to a case which was tried at the Central Criminal Court in October, 1843. (The Queen v. Haynes.) The prisoner had given to the deceased, the night before her death, two ounces of sulphate of potash, dissolved in water; and it was alleged that she had, a fortnight previously to this, taken, in divided doses, as much as a quarter of a pound of the salt. The woman supposed herself to be pregnant, which was disproved by an examination of the body; and it was charged that the prisoner had given her the salt with the intention of causing a miscarriage. After the last dose, she was seized with sickness, and died within a very short time. The stomach was found empty, but highly inflamed, and there was blood effused on the brain. One medical witness referred death to the action of this salt as an irritant poison; the other to apoplexy, as an indirect result of the violent vomiting caused by the salt. The prisoner was acquitted of the charge of murder, but subsequently found guilty of administering the drug with intent to procure abortion. Both of the witnesses admitted that, in small doses the salt was innocent; but that in the dose of two ounces it would produce dangerous effects. A portion of the sulphate in this case was examined by Mr. Brande, as it was suspected that some poisonous substance might have become accidentally mixed with it; but it was found to be pure.

It is not improbable, from the symptoms and the inflamed state of the stomach, that the salt acted here as an irritant poison; and the fact of its being an innocent medicine in small doses appears to be no sound objection to this view; for the same circumstance is observed with respect to many substances, the poisonous properties of which cannot admit of dispute. Some have ascribed the irritant properties of this and other saline medicines—such as cream of tartar, in large doses —to their insolubility, and to the fine spicula of the powdered salt acting mechanically upon the mucous membrane of the stomach. This explanation does not appear sufficient: 1st, because some of these saline medicines, when taken dissolved —such as alum and nitre—have had a similar action; and, 2d, the effects are very different, and far more rapidly fatal than in those cases where mechanical irritants—such as fine sand or iron filings—have been taken. In short, there is no doubt that if the same quantity of the salt were taken perfectly dissolved in water, it would have an equally irritant effect; and sulphate of potash has been known to act in this way, when taken in divided and therefore very soluble doses A case in which it thus proved fatal in *two hours,* is reported in the 'Annale d'Hygiène,' April, 1842. According to Mr. Mowbray (Medical Gazette, v. 33, p. 54), sulphate of potash is a salt much employed in France as a popular abortive. He quotes several instances in which, in large doses, it produced severe symptoms, resembling those of irritant poisoning, and even death. In one case, two drachms acted powerfully; and in another, that fell under his own observation, four drachms of the salt, administered to a lady after her confinement, had all the effects of an irritant poison. The case of Haynes is the first instance in which, I believe, it is publicly known to have proved fatal in England; and it shows that substances, commonly regarded as innocent, may give rise to important questions in toxicology.

NARCOTIC POISONS.

*Opium.* It has been frequently observed, in cases of poisoning by this drug, that the individual has recovered from the first symptoms, and has then had a relapse and died. There is some medico-legal interest connected with this state, which has been called secondary asphyxia from opium, although there appears to be no good reason for giving to it this name. In December 1843, a gentleman swallowed a quantity of laudanum, and was found labouring under the usual symptoms. The greater part of the poison was removed from the stomach by the pump; and he so far recovered from his insensibility, as to be able to enter into conversation with the surgeon; but a relapse took place, and he died the following night. It is not improbable that, in these cases, death may be occasioned by a portion of the poison which has been carried by the absorbents into the system.

*Recovery from a large dose without vomiting.* A case occurred at the Westminster Hospital, in December 1843, (Lancet, Dec. 1843,) in which a woman, ætat. 25, was brought into that Institution while labouring under the symptoms of poisoning by opium. She was perfectly comatose, the features devoid of expression, the lips purple, and the pupils contracted to the size of a pin's head. The eyes were everted and fixed. Sulphate of zinc and tartar emetic were given without effect, and the stomach-pump was not brought into use until about an hour after her admission. The contents of the stomach were entirely free from the smell of opium. The woman was kept roused, coffee was administered, and she recovered. It appears she had swallowed *one ounce of laudanum*, but at what time before her admission is not stated.

It is difficult to say on what the recovery of this woman depended, for a very long time had elapsed before the contents were removed from the stomach, and then there was no trace of opium to be perceived by the smell. A better plan for determining the presence of opium in the discharged liquid is to dilute it sufficiently, and observe whether it acquires a red colour with the sesquichloride of iron. This change is always produced where opium is present even in very small proportion, owing to the meconic acid which it contains. The test will act where no odour is perceptible, either from the quantity of the drug being too small, or its being concealed by other odours. It is certainly remarkable that this woman recovered, considering the largeness of the dose and the time which had elapsed before the stomach was evacuated.

*Dover's powder.* The following case of poisoning by Dover's powder has been reported by Mr. Griffiths, (Medical Gazette, March, 1844.) About ten grains of Dover's powder were given by mistake to an infant seven weeks old, and it died in twenty-four hours afterwards. The following is an account of the post-mortem appearances. The countenance was placid, and the fingers of both hands were firmly contracted. On opening the abdomen, the colon was seen to be distended with flatus; the spleen, kidneys, and intestines were healthy; the liver gorged with blood; the stomach contained a very small quantity of colourless viscid matter. The inner coat was vascular; and at the great curvature as well as in other parts were small patches of highly injected vessels. The lungs were gorged with blood; the upper lobes being infiltrated with a greenish serum. The pericardium was vascular, and contained about a drachm of fluid. The right auricle was empty; the left ventricle contained some thin fluid blood, and a small coagulum. The sinuses of the dura mater were filled with dark coagula; the surface of the brain appeared covered by a complete network of vessels, distended with light coloured blood. On the surface of each posterior lobe of the cerebrum, slight extravasation had taken place. The brain was soft, and the difference of colour between the gray and white matter barely discernible. The vessels in the substance of the brain were gorged with blood, presenting, on section, a thickly-studded appearance—the spots of a deep dull red, and in many places coalescing. There was a small quantity of fluid in each lateral ventricle, and on the floor of each, were large distended blood-vessels. There was serous effusion on the surface and at the base of the brain, to the amount of about half an ounce. The contents of the stomach were carefully analysed, but neither morphia nor meconic acid could be detected.

This case is interesting in several particulars. In the first place, it is surprising that so young an infant should have lived so long after taking a dose equivalent to one grain of opium. Making every allowance for the great vascularity of the brain in young subjects, it appears from the inspection, that the opium had here affected that organ, and caused a general congestion as well as effusion and slight extravasation, which last condition is somewhat rare in poisoning by opium. The non-detection of the poison in the contents of the stomach was sufficiently accounted for by the small quantity of opium in the Dover's powder, and by the length of time which the child survived. The opium contained in ten grains of Dover's powder is equivalent only to about the twentieth of a grain of morphia, and probably about the same proportion of meconic acid. It is extremely rare that opium is found in the stomachs of young children poisoned by small doses.

Dr. J. B. Beck has lately published, in the 'New York Journal of Medicine,' some excellent remarks upon the effects of opium on the infant subject. He shows that while this drug has a much greater effect on an infant in consequence of the greater impressibility of the nervous system, than on an adult, it is at the same time much more uncertain in its operation; and thus is liable to prove fatal in very small doses. Among the instances which he has accumulated, illustrative of the powerful action of the drug, he mentions one where a young child was narcotized by fifteen drops of paregoric elixir. This essay has been republished in the 'Medical Gazette,' for March, 1844, (vol. xxxiii, p. 767.)

*Quantity of opium required to destroy life.* The smallest quantity of opium in the solid state which has been known to destroy the life of an adult was four and a half grains mixed with camphor. This case is quoted by Dr. Christison. In September, 1843, an instance occurred in this metropolis of a woman, aged thirty-eight, being killed by eight grains of the drug given in two doses. These facts are interesting in a medico-legal point of view, by showing how small a quantity of this substance may, in some instances, destroy life.

*Solubility of opium in water.* So far as I am aware no experiments have been performed to determine the quantity of this drug taken up by water in the form of infusion. In November, 1843, a case of poisoning by opium was referred to me by Mr. T. O. Duke, of Kennington, in which the question arose. An ignorant nurse made an infusion by pouring hot water on powdered opium in a bottle, and gave, at short intervals, three teaspoonfuls of this infusion to a child aged about fourteen months, and it died poisoned by the drug in about eighteen hours. It was found that the infusion contained only 1·6 per cent. of solid matter, i.e. of the soluble part of the opium; and that the principal part of the meconate of morphia had been taken up, was proved by an infusion subsequently made, retaining only faint traces of that salt.

The results of some experiments on this subject were as follows: Fifteen grains of finely-powdered opium were infused, for twenty hours, with six drachms of boiling distilled water. On examination, the filtered infusion was found to contain 4 per cent. of solid matter, i.e. of the soluble part of opium. In another experiment, opium sliced was employed with water in the same proportions. The quantity dissolved averaged, on several trials, from 3 to 4 per cent. depending on the proportion of water and the length of contact. By boiling the residue in each case, a further quantity of meconate of morphia was obtained, showing that an aqueous infusion, while it will not extract the whole of the meconate at once, will yet take up sufficient to render it actively poisonous to young children.

*Prussic acid.* It has been a seriously debated question among medical jurists, whether an individual, after having swallowed a strong dose of prussic acid, could retain the power of performing certain acts indicative of volition and the preservation of sense. Two cases have occurred within the last year in England, which throw some additional light upon this important question, on which a charge of murder may sometimes depend. In one case, the deceased, an adult, swallowed three drachms of prussic acid from the phial in which it was contained, while another person was in the room with his back turned to him. This individual was alarmed by hearing the deceased exclaim "it's gone," and in answer to a question put by witness, said, "I have taken it." He was again about to speak, but his articulation failed him, he became insensible and died immediately afterwards.

The other case was referred to me from Suffolk, by Mr. Newham, surgeon of Bury St. Edmunds. In March, 1844, a commercial traveller was found dead in his bed at an inn. The evidence given at the inquest showed that he had died from the effects of prussic acid, and there could not be the slightest doubt that he had taken the poison himself. The point of interest connected with the case is, that when discovered dead, he was found lying on his left side in the natural position of rest, the legs being slightly drawn up to the abdomen; the arms bent over the chest; and although rigid, the hands were not clenched, nor did they appear in any way to have been spasmodically affected. The bed-clothes were smoothly drawn up to his shoulders, and there was no appearance whatever of disorder about them. On a chair beside the bed, at his back, was a phial holding about six drachms, and still containing a small portion of a liquid smelling strongly of prussic acid, mixed with the essential oil of lemons, which had probably been purposely mixed with it to disguise the odour. This phial was found with the cork in it. Mr. Newham correctly observes that this condition of things, clearly indicates a sequence of several voluntary acts performed by the deceased immediately before death; as, for instance, swallowing the acid from the bottle, then corking the bottle, placing it on a chair at the back of the bed, the turning over in bed, drawing up the bedclothes, and composing himself into a position of rest. From the evidence at the inquest, it appeared that not less than three drachms of prussic acid had been taken, and probably even a larger quantity; and the question arose, whether all the events above mentioned could have occurred between taking into the stomach so large a dose of this poison as to cause death without inducing convulsions, of which there were no signs? The fact that this was really a case of suicide, left it beyond doubt that the deceased had, after swallowing this dose, performed the series of acts above mentioned; and it was equally evident that convulsions had not taken place, at least so as to leave any sign of their existence in the dead body.

The reader will observe that this case is very similar in its details to that of Judith Buswell, for the alleged murder of whom, a young man named Freeman was tried at the Leicester Spring Assizes in 1829. (See Med. Gazette, vol. viii, p. 759.) The medical opinions in that case, from a similar series of acts, were rather against the presumption of suicide, and in favour of homicidal interference. It has been supposed, that when a strong dose of prussic acid destroys life so slowly as to give time for the performance of such voluntary acts, this would be indicated by the body being found in a convulsed state; when, on the other hand, death takes place so rapidly that there are no convulsions, then the inference should be that the deceased could not have retained sense or power sufficiently long for the performance of these acts. The above with other similar cases proves, that we cannot trust to an assumed criterion of this kind. There may be no mark of convulsion about a dead body,—circumstances may show that sense, volition, and a power of motion were actually retained for a certain period; and yet all this is compatible with the act being one of suicide from a large dose of prussic acid. We are not justified in inferring that a dose of this kind, when it operates slowly, is always and necessarily indicated by the body of the deceased being found in a convulsed state.

This question has acquired still greater interest from the late trial of Belany for poisoning his wife by prussic acid (Cent. Crim. Court, Aug. 1844.) The prisoner declared that the deceased shrieked, and afterwards told him that she had swallowed some "hot liquid." The medical witnesses are reported to have stated (although only from experiments on animals) that this shriek or cry was the immediate precursor of insensibility, and the last act of vitality,—in short that the power of speech would be then entirely lost. Hence the prisoner's statement would be inconsistent with truth. However strong the circumstantial evidence may have been against the accused, and it could scarcely have been stronger,—this medical opinion is not borne out by observation. In one instance, just related (p. 551), a larger dose of the poison was probably taken; but the deceased was able to answer a question and say, "*I have taken it*," before he became insensible. A very similar case, reported by Dr. Gierl, is to be found in most works on toxicology. These cases then clearly prove that, whether a shriek or cry be a *constant* accompaniment of poisoning by prussic acid or not,—a point which yet remains to be proved,—an individual may speak and even answer a question rationally after having taken the poison, and immediately before falling into a state of insensibility.

A remarkable trial has lately taken place at Chambèry, in which the accused was charged with the murder of the deceased by prussic acid; while, in the defence, it was alleged, that death was owing to apoplexy and not to the poison, (Annales d'Hygiène, 1843, p. 103.) The case presents numerous points of interest in relation to medico-legal toxicology; the symptoms and post-mortem appearances met with in apoplexy, as contrasted with those produced by prussic acid; the value of evidence derived from symptoms in cases of poisoning, as well as that obtainable from the period at which death ensues after the supposed administration; the extraordinary chemical errors that are occasionally made in the analysis of poisons, the witnesses in this case imagining that the presence of poison might be inferred from a series of very doubtful or even negative results. The person charged with the crime was very properly acquitted; for there was no medical proof whatever that poison had been the cause of death, while there was direct evidence of death from apoplexy, by the discovery of a large effusion of coagulated blood on the brain. He appears to have owed his acquittal principally to the care bestowed by Orfila, on the examination of the facts of the case.

*Oil of bitter almonds*. One case of poisoning by this substance has lately occurred, and is reported by Mr. Smith of Clifton, (Lancet, June, 1844.) A girl, between 8 and 9 years of age, swallowed about a teaspoonful of a mixture sold by druggists as "ratifia," composed of one part of the essential oil of bitter almonds to seven parts of spirit. The quantity swallowed by the patient was equivalent to about *seven drops* of the essential oil. With this datum it will be interesting to consider the effects produced by so small a dose. When seen immediately after the accident, there was complete insensibility; the eyelids were closed, but the eyes were brilliant and glassy, without any mental expression; the pupils dilated; no pulse at the wrist; the carotids beating fully and quickly; relaxation of the muscles of the extremities, but the lower jaw was clenched in rigid spasm. Cold affusion with stimulants, stimulating frictions and emetics, were employed. Vomiting was induced, and the ejecta had a strong smell of prussic acid. In about twenty minutes the pulse returned,—the child opened her eyes, and was able to answer questions.

The quantity of prussic acid contained in the oil, and to which its poisonous properties are due, is said to vary from 8 to 14 per cent. The above case shows that in a small dose it may give rise to very alarming symptoms; and it is probable, that but for the active and prompt treatment adopted this child would have died.

*Cyanide of potassium*. This salt has of late years caused death in several instances where it has been taken by mistake or in improper doses. A gentleman was killed in France, in 1843, by taking *twelve grains* of the salt, in consequence of some error in the medical prescription. The physician who ordered the medicine, was tried, fined and imprisoned. (Lancet, January, 1843.) Another case occurred at Breslau, in which a man, aged thirty, died in a *quarter of an hour* after taking a dose of a mixture which had been prescribed for him by his medical attendant, under all the symptoms of poisoning by prussic acid. (Henke's Zeitschrift der S. A., 1843, p. 7.) The mistake here arose from those unfortunate changes periodically made in the nomenclature of pharmacopœial compounds, which constitute a matter of regret among ourselves; for such a practice takes away all certainty from the art of prescribing, and leaves the life of the patient and the character of the practitioner in the hands of a druggist, who may be ignorant of the properties of the medicine which he dispenses.

It appears that until lately the yellow ferrocyanate of potash was known in the Prussian Pharmacopœia under the short name of "kali hydrocyanicum," just as it was formerly called, in English, prussiate of potash, and is now termed ferrocyanide of potassium—an objectionable alteration from the term ferrocyanate, because many dispensing druggists might confound the *ferro*cyanide with the cyanide, and dispense the poison for the innocent substance. Of late years, in the Prussian Pharmacopœia, the cyanide of potassium has received the name of "cyanetum kalicum," or, improperly, "kali hydrocyanium." *Fifteen grains* of "kali hydrocyanicum," in a dose, were prescribed by the physician for his patient, he meaning thereby the ferrocyanate of potash. Instead of this, however, cyanide of potassium was sent, and the patient died in a quarter of an hour. The physician adopted and employed the chemical name which was probably current at the time that he studied his profession. The party who dispensed the medicine was undoubtedly to blame; for it appears that he entertained some doubt about the largeness of the dose, and he ought to have known that a dose of such a compound could not be taken by a human being without certainly destroying life. The energy of the cyanide of potassium as a poison depends, in some measure, on its mode of preparation. Some specimens are so impure as to consist almost entirely of carbonate of potash, from which it may be separated by its ready solubility in alcohol. (See Annales d'Hygiène, 1843, p. 404, in which this subject is fully investigated by Orfila.) An opinion formerly prevailed, that the poisonous properties of the salt were destroyed under two circumstances: 1, by exposure to air, in which case it is transformed to carbonate of potash; and, 2, by its being heated, in solution, to the boiling point. In neither case, however, does the salt easily lose its poisonous properties. Orfila found that some which had deliquesced, by exposure to air for a fortnight, still acted as a poison; and the conversion of the salt, at 212°, into ammonia and formate of potash takes place so slowly, under the most favorable circumstances, as not to interfere with this poisonous action. This substance does not therefore become innocuous, as it was formerly alleged, by solution in *hot* water. I have found by experiment that the ebullition of a solution, continued for a quarter of an hour, produced no sensible quantity of formate of potash.

Accidents such as those above referred to often give rise to charges of malapraxis. A case occurred some years since on the continent, in which a physician prescribed three grains of the "murias hydrargyri" for a child. Calomel was then known by the termination "dulcis," and corrosive sublimate by the termination "corrosivus." The dispenser sent corrosive sublimate, and the dose killed the child. The physician was prosecuted for not having been more precise in his prescription; but it is fair to inquire whether a person who would in such a case send three grains of corrosive sublimate, to be taken by a child, was qualified for the dispensing of medicines under any circumstances whatever. Owing to the numerous changes that have taken place in our own Pharmacopœia, it is somewhat surprising that accidents have not occurred. Corrosive sublimate now differs from calomel merely in the prefix "bi," which might be in some cases overlooked. The impolicy of this change is apparent in the fact, that, on a new edition of the Pharmacopœia, if this system of adaptation to ephemeral chemical theories be adhered to, corrosive sublimate will become "chloride of mercury"—the name now attached to calomel; and this latter substance will become a

"dichloride." It is the opinion of some distinguished chemists, that what is commonly called peroxide is a protoxide of mercury, and the protoxide is a suboxide. All will agree that, for the safety of life, the names of medicines should be certain and unchangeable, and not vary with the fluctuating doctrines of the day; at any rate, it is a most serious result when the name attached to an innocent medicine at one time, should become applied to a powerful poison at another. Among the late "probability theories," as Berzelius terms them, which have emanated from the Giessen school, is one by which, if adopted, the present system of chemical and with it the pharmacopœial nomenclature will be completely overturned. Thus, an entirely new view is taken of the constitution of salts; and it is said that, instead of sulphate of potash being formed of an acid united to an alkaline base, it is the result of a union between a compound radical, formed of sulphuric acid and oxygen with the metal potassium. Pharmacy should be entirely independent of such hypothetical views; and all changes in the names of compounds should be made only for some very strong necessity, and with the greatest caution. It cannot be supposed that every practitioner throughout the empire should have the time, even if he had the inclination, to make himself master of the various speculations which are continually broached by chemists.

### NARCOTICO-IRRITANT POISONS.

*Cocculus indicus.* Some researches have been recently made by M. Chevallier on the effects of this powerful poison. (Annales d'Hygiène, 1843, p. 339.) It appears that it has been the practice, in some parts of France, to poison fish by a mixture of this substance with crumb of bread, and sell the fish for food; and it is stated that, in many instances, such fish were eaten without any ill effects resulting. This, however, was a matter of accident, and depended on the quantity of drug used; when this quantity was moderately large, the fish acted like a poison on animals. It would appear, from the observations of M. Goupil, that it is only the kernel of the berry which is poisonous, owing to the presence of picrotoxine,—that it is narcotico-irritant in its effects, and that the fish destroyed by it exert a similarly poisonous action when eaten. The woody shell of the berry is not poisonous—it merely operates as an emetic.

It appears that, with respect to this pernicious drug, the French system of legislation is like our own. There is a heavy penalty on the sale of it for certain purposes, but the free importation of it is allowed. The large quantities which are said to be openly and secretly imported into this country, can be applied to no lawful purpose; for the substance is utterly useless, both in medicine and the arts. There is no doubt that it is employed for the extensive adulteration of beer. The proper remedy would be to exclude it altogether; for it is absurd to attempt to prohibit its sale by a penalty, when its introduction has been once permitted.

*Cytisus laburnum.* The existence of a new and powerful narcotico-irritant poison has been lately announced by Dr. Christison, in the bark of the common laburnum tree. (Edin. Med. and Surg. Journ., Oct. 1843.) It is remarkable that, considering how widely this tree is diffused, and how accessible it is as a poison, as well as the fact that its noxious properties have been known for some time to the vulgar—at least in certain parts of the kingdom,—it has not before received any attention from toxicologists.

The case reported by Dr. Christison came to trial at the Inverness circuit last year. A youth, with the intention merely of producing vomiting in one of his fellow-servants, a female, put some dry laburnum-bark into the broth which was being prepared for their dinner. The cook, who remarked a "strong peculiar taste" in the broth, soon became very ill, and in five minutes was attacked with violent vomiting. The account of the symptoms is imperfect; for the cause of them was not even suspected until six months afterwards. The vomiting continued thirty-six hours; was accompanied by shivering,—pain in the abdomen, especially in the stomach,—and great feebleness, with severe purging. These symptoms continued, more or less, for a period of eight months; and she fell off in flesh and strength. At this period she was seen by a physician, who had been called on by the law-authorities to investigate the case. She was then suffering from gastro-intestinal irritation, vomiting after food, pain in the abdomen—increased by pressure, diarrhœa, tenesmus, and bloody stools, with other serious symptoms. The medical opinion was, that she was then in a highly dangerous state. The woman did not eventually recover until the following April. There was no doubt, from the investigation made by Dr. Ross and Dr. Christison, that her protracted illness was really due to the effects of the laburnum-bark.

Some experiments were then made on the action of the poison on animals. A teaspoonful of the powder of dry laburnum-bark was administered to a cat. Soon afterwards it writhed, apparently in great pain; in a short time it vomited violently, and, although languid and dejected for the rest of the day, it quickly recovered. Sixty-nine grains of the same powder were given to a dog. In ten minutes it whined and moaned, vomited violently, and soon got well. On a second occasion, twenty grains were found to act as a powerful emetic upon the animal. An ounce of the infusion of laburnum-bark, containing the active matter of sixty-two grains, was introduced by a catheter into the stomach of a full-grown rabbit. In ten minutes, the animal looked quickly from one side to the other, twitched back its head twice or thrice, and instantly fell on its side in violent tetanic convulsions, with alternating emprosthotonos and opisthotonos, so energetic, that its body bounded with great force upon the side, up and down the room. Suddenly, however, all movement ceased, respiration was at an end, the whole of the muscles became quite flaccid, no sign of sensation could be elicited, and the animal died within *two minutes and a half* after the poison was injected into the stomach. The body was opened in two minutes more, and the heart was found gorged, but contracting with some force. The stomach was filled with green pulp, soaked with the infusion. No morbid appearance was visible anywhere. In repeating this experiment, one rabbit died in half an hour, another in three quarters of an hour after small doses of the infusion were injected into the stomach; and a third rabbit speedily died, after eating greens merely impregnated with the infusion. In all these instances, convulsions were the leading symptoms produced. The same effects are popularly ascribed to the leaves, young pods, and seeds of the tree; but no experiments were performed with these.

The facts here detailed show that laburnum-bark is a most energetic poison—as powerful, even, as nux vomica. There are no means of detecting the nature of this poison, especially when administered in powder or infusion; or when, as in this criminal case, a decoction of the bark is given in food. The only plan for determining the deleterious properties of the substance, would be by exhibiting a portion to animals. As Dr. Christison remarks, these facts are of eonsiderable importance; and as they relate to a substance so common, and so easily obtained by every one, they ought to be more generally known to the profession than they are at present.

*Œnanthe crocata.* Another instance has occurred lately of the loss of life among the convicts at Woolwich, by the eating of the leaves and roots of this powerful indigenous vegetable poison. The facts have been communicated to the Medical Gazette, (May, 1844,) by Mr. Bossey. It appears that a party of convicts ate of the root and leaves of the plant while engaged at work. In about twenty minutes one man, without any apparent warning, fell down in strong convulsions, which soon ceased, but left a wild expression on his countenance. Soon afterwards, as many as nine fell into a state of couvulsions and insensibility. The face of the man first seized became bloated and livid; there was a sanguineous foam about the mouth and nostrils; the breathing was stertorous and convulsive; there was great prostration of strength, and insensibility: he died in *five minutes.* A second died, under similar symptoms, in a quarter of an hour, although the stomach-pump was used, and some leaves were extracted with the fluids. A third, who had assisted in carrying the two former, was himself seized with convulsions, and died in about an hour; and soon after him, a fourth died, in spite of the most energetic remedial treatment, by cold affusion, emetics, stimulants, stimulating frictions, and the use of the stomach-pump. Two other cases proved fatal, the one in nine days, and the other in eleven; and in these two cases, there was irritation of the alimentary canal. On inspecting the bodies of those who died quickly, there was congestion of the cerebral vessels, and, in one instance, a layer of extravasated blood was found beneath the pia mater. In the first case, which proved most quickly fatal, the cerebral vessels were not congested. The pharynx and œsophagus had a white appearance, contained some mucus and portions of the root. The lining membrane of the trachea and bronchi was intensely injected with dark blood. The lungs were gorged with fluid blood. The blood in the heart was very black and fluid. The stomach and intestines were externally of a pink colour: the cavity of the stomach was lined with a thick viscid mucus, containing portions of the root. The mucous membrane was much corrugated, and the follicles were particularly enlarged. Similar appearances were met with in all. In the two protracted cases, the mucous membrane of the stomach and bowels was softened and thickened. It had a pink colour externally, but no red appearance internally. The vessels of the brain were congested. In the others who partook of the roots, the symptoms were not so urgent. Under the free use of purgatives, considerable quantities of the root were discharged, and in a few days the men recovered. By a similar accident in 1834, the lives of four men were lost from the action of this vegetable poison.

There is no doubt that the œnanthe is one of the most powerful of the indigenous narcotico-irritant poisons. It destroys life with even greater rapidity than arsenic; for it here proved fatal to a strong healthy man in less than *one hour.* Chemists have not yet ascertained on what principle its active properties depend, but they appear to reside chiefly in the root.

*Digitalis Purpurea.* The following recent case of poisoning by this plant is reported by Mr. Wilson of Leeds (Med. Gaz., Aug. 1844.) A healthy robust young man, affected with sore throat, was advised to take "*throatwort tea.*" Having filled a quart pitcher with fresh leaves of the digitalis purpurea, he poured upon them as much boiling water as the pitcher would hold. Of this strong infusion he took a teacupful on going to bed which caused him to sleep soundly. In the morning he took a second cupful (the infusion being then much stronger), and went to his employment. He soon felt dizzy and heavy, began to stagger, lost his consciousness, and at length fell down in a state of syncope. On being conveyed home, he vomited severely and complained of extreme pain in the abdomen. When visited he was conscious, complained of great pain in his head,—the pupils were dilated, and the surface was cold, pallid, and covered with a copious perspiration. The pulse was low, about 40 in the minute,—three or four feeble pulsations being succeeded by a complete intermission of several seconds; and each stroke, though weak, was given with a peculiar "explosive shock." There was still great pain in the abdomen, with incessant and violent vomiting, no diarrhœa,—suppression of urine, and an abundant flow of saliva. Brandy and ammonia with warmth were employed, and after reaction had commenced,—purgatives were administered. The man slowly recovered, but the pulse presented its peculiar beat and weakness for several days; and during this time, the man could not bear the upright position.

The symptoms in this case were like those which have been usually observed. It establishes beyond question that salivation may be produced by this plant.

*Alcohol.* A singular instance is referred to in a late number of the 'Lancet' (April, 1844), in which a child aged 2 years was thrown into an apoplectic stupor, from the alcoholic vapour of eau de Cologne. There is no doubt that the long-continued respiration of the vapour of alcohol or ether might prove dangerous to a child.

